# Tracing founder haplotypes of Japanese apple varieties: application in genomic prediction and genome-wide association study

**DOI:** 10.1038/s41438-021-00485-3

**Published:** 2021-03-01

**Authors:** Mai F. Minamikawa, Miyuki Kunihisa, Koji Noshita, Shigeki Moriya, Kazuyuki Abe, Takeshi Hayashi, Yuichi Katayose, Toshimi Matsumoto, Chikako Nishitani, Shingo Terakami, Toshiya Yamamoto, Hiroyoshi Iwata

**Affiliations:** 1 Laboratory of Biometry and Bioinformatics, Department of Agricultural and Environmental Biology, Graduate School of Agricultural and Life Sciences, The University of Tokyo, 1-1-1 Yayoi, Bunkyo, Tokyo, 113-8657 Japan; 2 Institute of Fruit Tree and Tea Science, National Agriculture and Food Research Organization (NARO), 2-1 Fujimoto, Tsukuba, Ibaraki 305-8605 Japan; 3 Division of Apple Research, Institute of Fruit Tree and Tea Science, NARO, 92-24 Shimokuriyagawa Nabeyashiki, Morioka, Iwate 020-0123 Japan; 4 Institute of Crop Science, NARO, 2-1-2 Kannondai, Tsukuba, Ibaraki 305-8518 Japan; 5 Institute of Agrobiological Sciences, NARO, 1-2 Owashi, Tsukuba, Ibaraki 305-8634 Japan

**Keywords:** Plant breeding, Plant breeding

## Abstract

Haplotypes provide useful information for genomics-based approaches, genomic prediction, and genome-wide association study. As a small number of superior founders have contributed largely to the breeding history of fruit trees, the information of founder haplotypes may be relevant for performing the genomics-based approaches in these plants. In this study, we proposed a method to estimate 14 haplotypes from 7 founders and automatically trace the haplotypes forward to apple parental (185 varieties) and breeding (659 F_1_ individuals from 16 full-sib families) populations based on 11,786 single-nucleotide polymorphisms, by combining multiple algorithms. Overall, 92% of the single-nucleotide polymorphisms information in the parental and breeding populations was characterized by the 14 founder haplotypes. The use of founder haplotype information improved the accuracy of genomic prediction in 7 traits and the resolution of genome-wide association study in 13 out of 27 fruit quality traits analyzed in this study. We also visualized the significant propagation of the founder haplotype with the largest genetic effect in genome-wide association study over the pedigree tree of the parental population. These results suggest that the information of founder haplotypes can be useful for not only genetic improvement of fruit quality traits in apples but also for understanding the selection history of founder haplotypes in the breeding program of Japanese apple varieties.

## Introduction

Apple is a widely consumed fruit, with well-known health benefits^1^. The popular adage “an apple a day keeps the doctor away” showcases this idea. Approximately 86 Mt of apple fruit was produced worldwide in 2018 (FAOSTAT, http://faostat.fao.org). Due to the importance of this fruit as a horticultural crop, the genomic information of apples has been vigorously developed. The first reference whole-genome sequence of the apple cultivar ‘Golden Delicious’ was released in 2010 (ref. ^2^), which accelerated the scientific interest in apples. The report has been cited almost 1000 times in the 8 years since its publication^3^. Three systems of single-nucleotide polymorphism (SNP) genotyping arrays, the Illumina 8K^4^ or 20K^5^, or the Affymetrix Apple480K^6^ arrays have been developed for apples. In addition to SNP genotyping arrays, other methods, such as genotyping-by-sequencing (GBS) using next-generation sequencing (NGS), have been developed and applied in apples^7–10^.

The SNP data collected with the genotyping systems have been used to perform genomic prediction (GP) and genome-wide association study (GWAS) in apples^7–13^. GP and GWAS are genomics-based approaches for plant breeding and genetics, which are especially useful in fruit trees because of their perennial nature and long generation time^14^. GP is the prediction of genomic estimated breeding values (GEBV) based on genome-wide markers. GEBV is used for the selection of superior individuals, which is called genomic selection (GS)^15^. GS has greater potential than marker-assisted selection (MAS), especially for complex traits controlled by a large number of genes, such as fruit quality traits^16,17^. GWAS offers advantages over traditional bi-parental quantitative trait loci (QTL) mapping, as GWAS does not require the maintenance of a large segregating population^18^. The accuracy of GP and the resolution of GWAS depend on the degree of linkage disequilibrium (LD) between the SNPs and QTL^16–18^.

GP and GWAS using haplotype information consisting of multiple SNPs have become popular because the marker density is higher. Even when individual SNPs are not in complete LD with a QTL, haplotypes may be in complete LD with the QTL by using clusters of related SNPs as a haplotype marker^19^. The optimal methods to form haplotype blocks have been discussed for GP^20,21^ and GWAS^22,23^. The haplotype-based GWAS may solve synthetic associations, where rare SNP variants have strong LDs with many other non-causative rare SNP variants^18^. Compared to SNPs, the use of haplotype information improves the accuracy of GP in dairy cattle^24^ and maize^25^ and the resolution of GWAS in barley^26^ and soybean^27^. The superiority of GP and GWAS focused on the use of ancestral haplotypes compared with SNPs has also been reported in dairy cattles^28,29^. The use of founder haplotypes may be worthwhile in performing GP and GWAS, especially for fruit trees as most of its breeding programs have used a small number of superior founders^30,31^, compared with cereal crops. However, the potential of GP and GWAS using founder haplotype information has not been investigated in fruit trees. Haplotype information has also been used to survey the pedigree of important cultivars of apple. Howard et al. elucidated the unknown parent and grandparents of an important apple cultivar, ‘Honeycrisp’, through haplotype analysis of grandparents^32^. Kunihisa et al. traced the haplotypes of another important apple cultivar, ‘Fuji’, in its relatives, to develop markers for the selection of the preferable phenotypes related to ‘Fuji’^31^. However, these manual methods of assigning and tracing the haplotypes for each individual are laborious and time-consuming.

Most of the apples bred in Japan mainly originate from only seven founders: ‘Ralls Janet’, ‘Delicious’ strains, ‘Golden Delicious’, ‘Jonathan’, ‘Worcester Pearmain’, ‘Indo’, and ‘Cox’s Orange Pippin’^31^. In this study, we proposed a method to automatically assign 14 haplotypes of the 7 founders to the whole-genome region of apple parental and breeding populations by combining multiple algorithms based on genealogical information tracing local haplotypes of individuals back to the founder haplotypes. We validated the method by a comparison with the manual method described by Kunihisa et al.^31^. The objective of this study was to evaluate the performance of GP and GWAS based on founder haplotype information. Moreover, we aimed to visualize and reveal the transmission of these haplotypes in the parental population to understand the selection history of founder haplotypes in the breeding program of Japanese apple varieties.

## Results

### Tracing founder haplotypes forward to apple parental and breeding populations

**Fig. 1:**
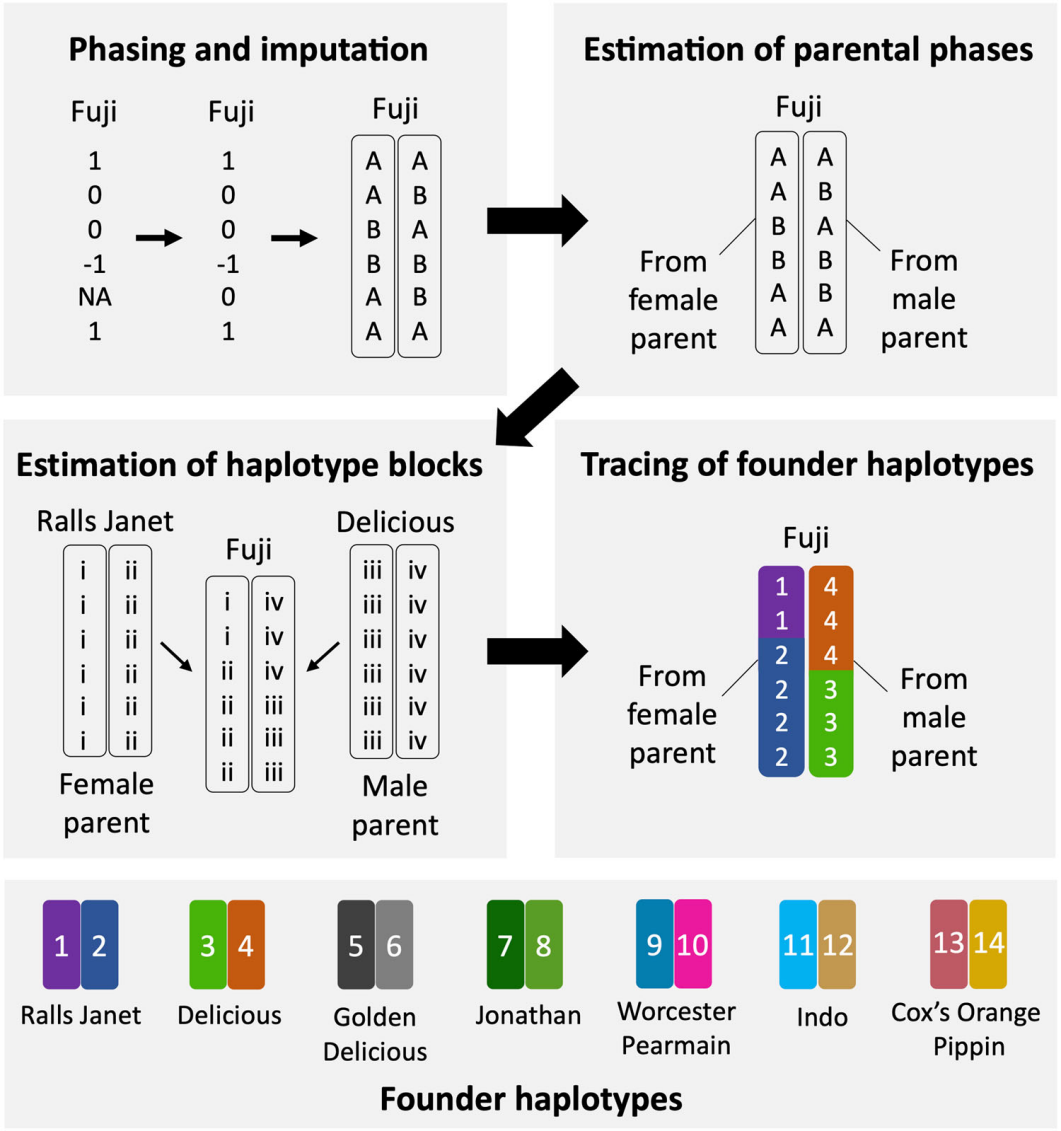
Scheme for tracing the haplotypes of founder cultivars. Fourteen founder haplotypes were traced automatically forward to a total of 844 individuals from the combined populations, according to four steps. A famous cultivar ‘Fuji’ is shown as an example. The first step is the phasing and imputation of sporadic missing genotypes of ‘Fuji’. The second step is the estimation of its parental phases. The third step was the estimation of haplotype blocks of ‘Fuji’ inherited from the female ‘Ralls Janet’ and male ‘Delicious’ parents. The final step was to assign the founder haplotypes of ‘Ralls Janet’ and ‘Delicious’ to the haplotype blocks of ‘Fuji’.

The 14 haplotypes of 7 founder cultivars were automatically estimated and traced forward to a total of 844 individuals from the parental and breeding populations (Supplementary Tables S1 and S2) by the four steps indicated in Fig. 1. The Beagle method showed superior phasing accuracy (0.98) (Table 1), compared with other methods and the manual method that was assumed to provide correct phasing. The accuracy of founder haplotyping using the most accurate Beagle-phasing data was the highest (0.90) among the methods; however, findhap.f90 showed the lowest accuracy of founder haplotyping, although it showed the second highest phasing accuracy among the methods (Table 1). Finally, 92% of the Beagle-phasing SNP data of the combined population (parental and breeding populations) could be represented by the information of the 14 founder haplotypes. Conversely, 86% of the manual method SNP data could be represented by the information of the founder haplotypes.

**Table 1:** Accuracy of phasing and haplotyping based on four software algorithms.

	Beagle	findhap.f90	MaCH	fastPHASE
Phasing accuracy	0.98	0.97	0.97	0.94
Haplotyping accuracy	0.90	0.68	0.87	0.69

### Accuracy of GP based on founder haplotype information

A high degree of LD was retained even at longer distances between SNPs; for example, 0.19 and 0.11 at 1 Mb and 3 Mb, respectively (Supplementary Fig. S1). The mean LD between adjacent SNPs in the combined population was 0.33 (56.97 kb). Principal component analysis (PCA) showed no clear genetic differentiation in the parental and combined populations, although small clusters of each family were observed (Supplementary Fig. S2). A similar result was observed in hierarchical clustering and ADMIXTURE clustering (Supplementary Figs. S3 and S4).

**Fig. 2:**
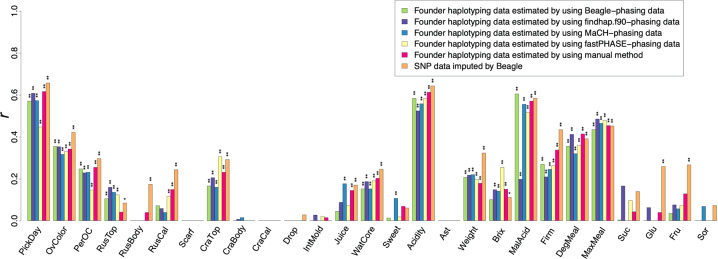
Accuracy of genomic prediction in the breeding population. Prediction accuracy was evaluated using the Pearson’s correlation coefficient (*r*) between the predicted genotypic values and phenotype-observed values. Asterisks indicate statistically significant correlations: *p < 0.05; **p < 0.01.

Six GP methods were compared for 27 fruit quality traits (Table 2 and Fig. 2). Higher accuracy was observed using the founder haplotypes estimated from Beagle- or findhap.f90-phasing data than that from MaCH- and fastPHASE-phasing data for 9 of the 12 traits, where all 4 models showed significant correlations in the test of no correlation. The models using automatically estimated founder haplotypes outperformed the model using manually estimated data in 7 of the 14 traits, where both models based on automatically and manually estimated founder haplotypes showed significant correlations in the test of no correlation. The use of automatically or manually estimated founder haplotypes improved the accuracy compared to the use of SNPs in 7 of the 15 traits, where both models based on founder haplotypes and SNPs showed significant correlations in the test of no correlation. However, in the other eight traits, the results were opposite. The prediction accuracy was relatively high in harvest time (PickDay), acidity (Acidity), and malic acid (MalAcid) (*r* ≥ 0.6).

**Table 2:** Fruit quality traits analyzed in this study.

Trait	Abbreviation	Description [assessment type]	Evaluation year			
			Parental pop.	Breeding pop.		
				‘Orin’ × ‘Akane’	‘Shinano Gold’ × Morioka 64 ‘Sinsekai’ × ‘Morinokagayaki’ ‘Kotaro’ × ‘Kinshu’	Others
Harvest time	PickDay	Number of days to harvest from January 1	1–25	3	1–3	1–4
Over color	OvColor	Fruit skin redness. Rank: 1 (green/yellow/orange), 2 (pale red), 3 (red/brown), 4 (bright red/purple), 5 (deep red) [visual]	1–25	3	1–3	1
Degree of skin coloration	PerOC	Proportion of fruit skin area coloring red. Rank: 1 (<20%), 2 (20≤–<40%), 3 (40%≤–<60%), 4 (60%≤–<80%), 5 (80%≤) [visual]	1–25	3	1–3	1
Russet top	RusTop	Fruit skin area covered with russet, examined from the top side. Rank: 1 (nil), 2 (little), 3 (mild), 4 (large), 5 (whole) [visual]	1–25	3	1–3	1
Russet body	RusBody	Fruit skin area covered with russet, examined from the horizontal side. Rank: 1 (nil), 2 (little), 3 (mild), 4 (large), 5 (whole) [visual]	1–25	3	1–3	1
Russet calyx	RusCal	Fruit skin area covered with russet, examined from the calyx side. Rank: 1 (nil), 2 (little), 3 (mild), 4 (large), 5 (whole) [visual]	1–25	3	1–3	1
Scarf skin	Scarf	Presence of scarf skin on fruit. 0 (absence), 1 (presence) [visual]	1–25	3	1–3	1
Cracking top	CraTop	Presence of fruit cracking, examined from the top side. 0 (nil), 1 (observed) [visual]	—	2	1–3	1
Cracking body	CraBody	Presence of fruit cracking, examined from the horizontal side. 0 (nil), 1 (observed) [visual]	—	2	1–3	1
Cracking calyx	CraCal	Presence of fruit cracking, examined from the calyx side. 0 (nil), 1 (observed) [visual]	—	2	1–3	1
Preharvest fruit drop	Drop	Degree of fruit drop before the harvest time. Rank: 1 (nil), 2 (slight), 3 (moderate), 4 (severe) [visual]	2	2	1–3	1
Internal mold	IntMold	Presence of mold in fruit core. 0 (nil), 1 (observed) [visual]	—	3	1–3	1
Juiciness	Juice	Juiciness during chewing after peeling. Rank: 1 (dry), 2 (slightly dry), 3 (intermediate), 4 (slightly juicy), 5 (juicy) [sensory]	1–25	3	1–3	1
Degree of watercore	WatCore	Degree of watercore observed in equatorial plane of fruit. Rank: 1(nil), 2(slight), 3(moderate), 4(severe) [visual]	1–25	3	1–3	1
Sweetness	Sweet	Sweetness of peeled fruit. Rank: −2 (weak), −1 (rather weak), 0 (moderate), 1 (rather strong), 2 (strong) [sensory]	1–25	3	1–3	1
Acidity	Acidity	Acidity of peeled fruit. Rank: −2 (weak), −1 (rather weak), 0 (moderate), 1 (rather strong), 2 (strong) [sensory]	1–25	3	1–3	1
Astringency	Ast	Astringency of peeled fruit. 0 (absence), 1 (presence) [sensory]	—	—	1–3	1
Weight	Weight	Fruit weight (g)	1–25	3	1–3	1
Soluble solid content	Brix	Brix of the squeezed juice, measured by a refractometer	1–25	2	—	1
Malic acid	MalAcid	Acidity of squeezed juice (%), measured as titratable acid content converted into malic acid weight	1–25	2	—	1–2
Firmness	Firm	Mean firmness of sunny and shaded sides of the fruit (Magness-Taylor penetrometer) (lb)	1–25	2	—	1–2
Degree of mealiness	DegMeal	Flesh mealiness after storage for 28–30 days under 20 °C, measured following to Moriya et al.^75^	2	2	—	1
Max of mealiness	MaxMeal	Flesh mealiness after storage for 40 days under 20 °C	—	—	—	1
Sucrose	Suc	Sucrose content of the juice (mg/mL), obtained using a high-performance liquid chromatograph (HPLC)	2	1	—	—
Glucose	Glu	Glucose content of the juice (mg/mL), obtained using an HPLC	2	1	—	—
Fructose	Fru	Fructose content of the juice (mg/mL), obtained using an HPLC	2	1	—	—
Sorbitol	Sor	Sorbitol content of the juice (mg/mL), obtained using an HPLC	2	1	—	—

### GWAS and propagation of founder haplotypes

**Fig. 3:**
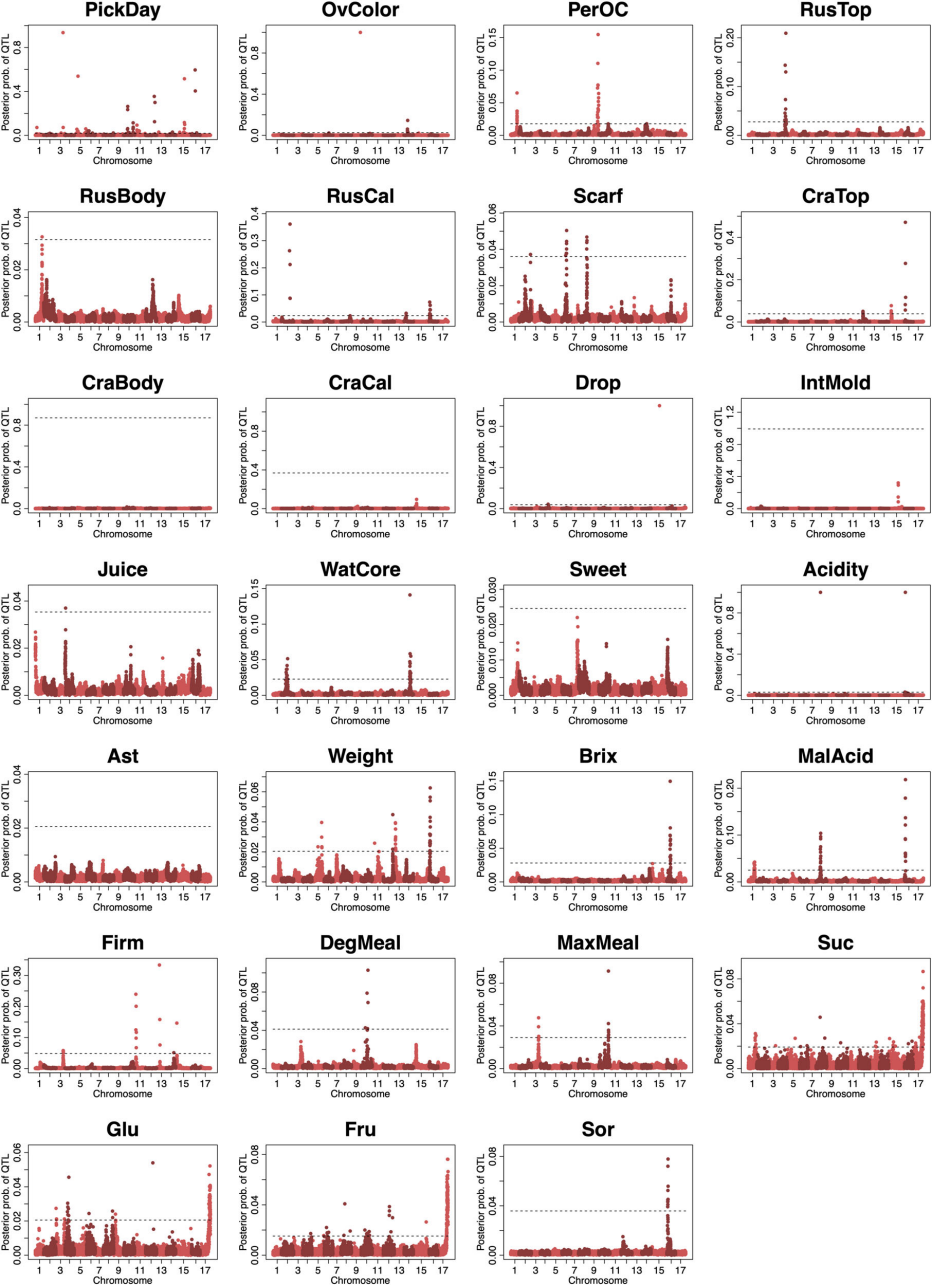
GWAS based on founder haplotypes in the combined population. Dashed lines indicate the significant thresholds obtained from the random permutation analysis.

GWAS was carried out for the 27 fruit quality traits (Table 2) using the combined population (Fig. 3, Supplementary Fig S5, and Supplementary Tables S3 and S4). GWAS based on founder haplotypes showed broader peaks in the significant regions than GWAS based on SNPs. Significant associations were detected in the same 22 traits between GWAS based on founder haplotypes and SNPs. In 21 out of 22 traits (except juiciness [Juice]), the common chromosomes of significant associations were detected; for example, Chr. 9 for the degree of skin coloration (PerOC) and Chr. 14 for the degree of watercore (WatCore). Some significant associations were detected only in GWAS based on founder haplotypes in 13 traits, including in Chr. 1 for PerOC and Chr. 2 for WatCore. Conversely, some significant associations were detected only in GWAS based on SNPs in 16 traits, for example, in Chr. 8 and 14 for PerOC. The genetic effects of the founder haplotypes at the significant loci for each trait could also be estimated by GWAS based on founder haplotypes (Supplementary Table S3). Founder haplotype 10 (one of ‘Worcester Pearmain’) showed the largest effect in PerOC at the most significant locus on Chr. 9. On the contrary, founder haplotype 12 (one of ‘Indo’) showed the smallest effect on the trait at the locus. For WatCore, founder haplotype 4 (one of ‘Delicious’) and 6 (one of ‘Golden Delicious’) showed the largest or smallest effects, respectively, at the most significant locus on Chr. 14. However, founder haplotype 4 (one of ‘Delicious’) showed the smallest effect for the trait at the most significant locus on Chr. 2. The contribution of the most significant locus on Chr. 14 was higher than that on Chr. 2 for the trait (Supplementary Table S3).

The R2R3 MYB transcription factor gene, *MdMYB1*^33^, was found in the significant region on Chr. 9 for PerOC in GWAS based on founder haplotypes and SNPs (Supplementary Fig. S6). The gene was located at a distance of 1.8 Mb from the most significant locus in the GDDH13 genome. This gene is a key regulator of apple skin color^33^. Two other genes, *MD09G1265100* and *MD09G1278400*, which were annotated as R2R3 MYB transcription factor genes, were found at a distance of 26 kb and 1.6 Mb, respectively, from the most significant locus. In the significant region on Chr. 1 for PerOC in GWAS based on founder haplotypes, three genes, *MD01G1155100*, *MD01G1176700*, and *MD01G1200200*, were located within 2 Mb of the most significant locus (Supplementary Fig. S6). *MD01G1155100* and *MD01G1200200* were annotated as R2R3 MYB transcription factors, whereas *MD01G1176700* was annotated as an MYB-like transcription factor.

Seven genes, *MD14G1159400*, *MD14G1160300*, *MD14G1160600*, *MD14G1160700*, *MD14G1161400*, *MD14G1161700*, and *MD14G1161800*, were found within 1.5 Mb of the most significant locus on Chr. 14 for WatCore in GWAS based on founder haplotypes and SNPs (Supplementary Fig. S7). *MD14G1161700* and *MD14G1161800* were annotated as malate synthases, and the other five genes were annotated as β-glucosidases. *MD02G1161700*, annotated as an ethylene receptor, was located in the significant region on Chr. 2 for WatCore in GWAS based on founder haplotypes (Supplementary Fig. S7).

**Fig. 4:**
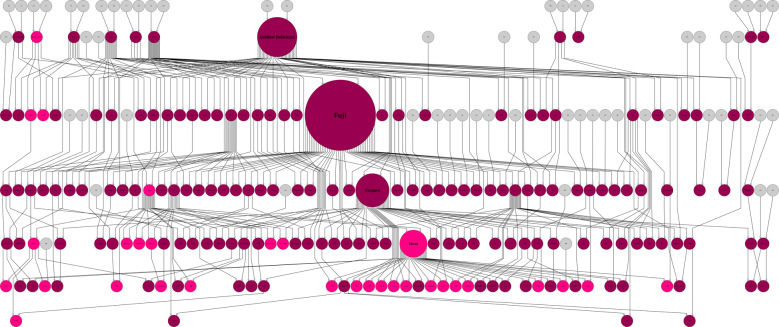
Propagation of founder haplotype 10 (one of ‘Worcester Pearmain’) in the parental population. The right pink indicates the cultivar or breeding line with the founder haplotype 10 (one of ‘Worcester Pearmain’), which showed the largest PerOC effect in the most significant marker locus on Chr. 9. The deep pink indicates the other cultivar or breeding line used in this study. Unknown parents are indicated by gray. The circle size was based on pedigree contribution.

Random transmission test of founder haplotypes revealed that the frequency of the founder haplotype 10 (one of ‘Worcester Pearmain’) on Chr. 9, which showed the largest effect on PerOC, was significantly increased in the parental population (Fig. 4 and Supplementary Table S3). In the progenies of ‘Sansa’, ‘Akane’, and ‘Chinatsu’, 56, 69, and 75% of them had the founder haplotype 10 (one of ‘Worcester Pearmain’), respectively (Fig. 4). In contrast, the frequency of the founder haplotype 5 (one of ‘Golden Delicious’), which showed the smallest effect in the trait at the second significant locus on Chr. 1, was significantly decreased in the parental population (Supplementary Table S3). The frequency of the founder haplotype 6 (one of ‘Golden Delicious’) on Chr. 14, which showed the smallest effect in WatCore, was significantly increased in the parental population.

## Discussion

In this study, we proposed a method for automatically tracing founder haplotypes forward to a population by combining multiple algorithms. We applied the method to the apple parental and breeding populations and traced the 14 haplotypes of the 7 founders forward to a total of 844 individuals of the population. Overall, 92% of SNPs in the combined population could be assigned based on the information of the 14 founder haplotypes. This method may be applicable to other fruit tree species, whose breeding programs have employed a limited number of founders (e.g., citrus^30^ and peach^34^). Beagle and findhap.f90 attained similar or higher phasing accuracy compared to MaCH and fastPHASE, suggesting that using both LD and pedigree information is more effective for phasing than using LD information only. The importance of using both LD and pedigree information was also demonstrated as the higher imputation accuracy in a study of dairy cattle^35^. The highest phasing accuracy of Beagle may have resulted in the highest founder haplotyping accuracy among the methods. These results suggested that Beagle was the best method to trace founder haplotypes. However, findhap.f90 showed the lowest accuracy of founder haplotyping, despite having the second highest phasing accuracy among the methods. The possibility that the manual method may contain some errors cannot be excluded, taking into consideration the higher GP accuracy of findhap.f90 in over color (OvColor), weight (Weight), and max of mealiness (MaxMeal) traits, compared to the manual method.

Recently, a large SNP genotyping array (Affymetrix Apple480K^6^) was developed for apples. The array was built by resequencing 63 different cultivars covering most of the genetic diversity in cultivated apples (*Malus* × *domestica*), whereas the Illumina 20K^5^ used in this study was built by resequencing 13 apple cultivars (*M*. × *domestica*) with one accession belonging to a crab apple species (*M*. *micromalus*). The accuracy of founder haplotyping may be improved by using such high-density SNP arrays covering broader apple cultivars. An NGS-based GBS method would also be a good way to obtain denser SNP information. However, the GBS method has the drawback of a large amount of missing data because of the limited sequencing depth^36^.

The high LD and strong genetic relation observed in the combined population may be a result of a population bottleneck^37^ caused by the limited number of founders. The mean *r*^2^ value between adjacent SNPs in the combined population (0.33) was slightly higher than that in another apple population of New Zealand (0.32)^11^ and in strawberry (0.26)^38^ but lower than in citrus (0.45)^39^ or Japanese pear (0.34)^40^. Strong genetic relations have also been reported in citrus^39^ and Japanese pear^40^, in which populations were generated by a limited number of founders. A strong subpopulation structure, such as that found between *indica* and *japonica* rice varieties^41^, can cause spurious associations in GWAS^18^. Selecting a population that is not genetically highly structured and inter-related yet exhibits high phenotypic diversity is important for efficient GWAS^42^. The high LD pattern and the absence of a clear subpopulation structure observed in this study would be a good key for the success of GP^43,44^ and GWAS^18^.

Among the GP methods, the model based on the automatic assignment of founder haplotypes showed a higher prediction accuracy than the model based on the manual assignment in seven traits, suggesting that the automatic assignment is a useful alternative to the manual assignment. The model based on automatic or manual assignment of founder haplotypes outperformed the model based on SNP genotypes in seven traits, implying that the founder haplotypes may have higher LD with the QTL than SNPs. Cuyabano et al.^24^ suggested that haplotypes can better capture mutations in more than one locus from the result of the better performance of haploblock for GP compared with SNPs. The allogamous apple population used in this study could also contribute to the superiority of GP using founder haplotypes. Matias et al.^25^ showed that the use of haplotype matrices increases the ability of GP in a maize breeding population; however, this was not observed in a rice population. This result indicated that the use of haplotypes has the potential to increase the ability of GP in allogamous plants.

In contrast, the models based on automatic and manual assignment of founder haplotypes were inferior to the model based on SNP genotypes in eight traits. This result implies that the number and length of haplotypes defined as founder haplotypes in this study could not always be suitable for GP. The parental and breeding populations obtained through several crosses from the founder cultivars were thought to consist of long blocks of the founder haplotypes. The founder haplotypes in the long blocks may not always represent substantial variations related to the traits. Hess et al.^21^ revealed that GPs are more accurate with short haplotypes or less accurate with long haplotypes (>500 kb) than with SNPs, in a study using an admixed dairy cattle population. In addition, the optimum haplotype length is reported to depend on the traits^20^. To improve GP accuracy, we will need to consider the optimum number and length of haplotypes for each trait, not founder haplotypes defined in this study. Calus et al.^45^ showed that the benefit of haplotype analysis is decreased at high SNP marker density. This is because SNPs are expected to be closely linked to some important QTL at higher marker density. The density of SNP markers used in this study may be sufficient for GP.

The prediction accuracy was relatively high in PickDay (*r* ≥ 0.6), where significant loci were detected across the genome by GWAS, suggesting that GS, not MAS, may be useful for the trait. Compared with other studies of fruit trees (e.g., citrus^39^ and Japanese pear^40^), the moderate or lower prediction accuracies (*r* < 0.7) for all the traits observed in this study, which were consistent with previous reports (e.g., over color and russet^46^), could be caused by the lower mean LD value (0.33) compared with that in citrus (0.45)^39^ and Japanese pear (0.34)^40^.

In GWAS based on the assigned founder haplotypes, we detected significant associations in 22 traits and estimated the genetic effects of the haplotypes at the significant loci. We visualized the transmission of the founder haplotype with the largest genetic effect over the pedigree tree of the parental population. The visualization enables us to understand the selection history of the loci, which may be difficult to determine with SNP genotypes alone. The founder haplotypes may be useful as markers for haplotype-based breeding^47^, in which superior haplotypes of the target traits are assembled to improve crops.

Significant associations were detected only in GWAS based on founder haplotypes in 13 traits, implying that QTL related to the traits could represent more than one polymorphism. The power of haplotype markers was greater when a QTL was due to more than one polymorphism in a coalescent simulation study^22^. GWAS based on founder haplotypes could decrease the influence of synthetic associations^18^ by using the clusters of SNPs as founder haplotypes. The bottleneck of a population caused by a limited number of founders could also provide the advantage of GWAS based on founder haplotypes. Hamblin and Jannink^22^ showed that the power of 2- and 3-SNP haplotype markers to detect a QTL exceeds that of single-SNP markers, and this is particularly relevant to the population that experienced bottlenecks in the simulation study. In 16 traits, some significant associations were detected only in GWAS based on SNP genotypes, implying that GWAS based on founder haplotypes and based on SNP genotypes is worthwhile to detect the QTL because one QTL may be detected with one method but not the other, as suggested by Hamblin and Jannink^22^.

The most significant association was detected on Chr. 9 for PerOC and was consistent with associations detected previously^7,11–13,48,49^. The three R2R3 MYB transcription factor genes were found in the significant region on Chr. 9. One of the three genes, *MdMYB1*, has been reported to be a key regulator of anthocyanin accumulation^33^. Red coloration on the skin of apples is one of the major interests for breeders and consumers. *MdMYB1-1* is the only dominant allele associated with the red skin color; conversely, *MdMYB1-2* and *MdMYB1-3* are non-coloring (i.e., yellow or green) alleles. The founder haplotype 10 (one of ‘Worcester Pearmain’), which showed the largest effect for PerOC in the most significant locus on Chr. 9, had *MdMYB1-1* allele^13^, while the founder haplotype 12 (one of ‘Indo’), which showed the smallest effect in the locus, had *MdMYB1-2* allele^13^; that is, these founder haplotypes had a similar positive or negative effect for PerOC and this was related to *MdMYB1*. The significant increase of the frequency of the founder haplotype 10 (one of ‘Worcester Pearmain’) in the parental population and the high incidence (>50%) of the founder haplotype 10 in the progeny of ‘Sansa’, ‘Akane’, and ‘Chinatsu’ indicate that the founder haplotype 10 (one of ‘Worcester Pearmain’) may have been positively used by breeder to select the red skin apple, one of the selection criteria. The *MdMYB1* locus has been reported to explain >80% of the phenotypic variance for apple skin color^3^; however, the ﻿causative locus to explain the remaining 20% of the phenotypic variance remains unclear. As discussed by Kunihisa et al.^31^, the depth of red color also varied among the apple varieties with homo *MdMYB1-1* alleles in this study. Another significant association for PerOC was detected on Chr. 1 in GWAS using founder haplotypes, which was consistent with another GWAS study by McClure et al.^49^. Two R2R3 MYB transcription factor genes, as well as one MYB-like transcription factor gene, were found within 2 Mbp from the most significant locus on Chr. 1. A MYB-like transcription factor gene, *Peace*, reportedly controls anthocyanin coloration in the flower of peach^50^. R2R3-MYB transcription factors are known to be key regulators of skin color in apple^33^ and peach^51^. The frequency of the founder haplotype 5 (one of ‘Golden Delicious’), which showed the smallest effect for PerOC in the second significant locus on Chr. 1, was significantly decreased in the parental population, suggesting that the founder haplotype 5 (one of ‘Golden Delicious’) may be removed for the selection of the red skin apple by breeders. Thus the significant locus on Chr. 1 could be another candidate for PerOC control.

Significant associations with WatCore were detected on Chr. 2 and 14. The association detected on Chr. 14 was previously reported by Kunihisa et al.^31^. The haplotype of ‘Delicious’ on Chr. 14 dominantly caused watercore, and the other haplotype, not ‘Delicious’ or ‘Ralls Janet’, hardly caused watercore in the study using ‘Fuji’ and its relatives^31^. The founder haplotype 4 (one of ‘Delicious’) showed the largest dominant effect on Chr. 14 also in this study. Strains of the ‘Delicious’ cultivar are well known to be highly susceptible to watercore, which is prone to several physiological disorders, such as browning and breakdown during storage; therefore, watercoring in apples can be viewed negatively^52^. The significant increase in the frequency of the founder haplotype 6 (one of ‘Golden Delicious’) on Chr. 14, which showed the smallest effect for WatCore, implies that the founder haplotype 6 (one of ‘Golden Delicious’) has been used for the selection to remove the undesired watercores. However, watercored apples (e.g., ‘Fuji’) have been gradually welcomed by Asian countries in the past decade^52^. Thus the founder haplotype 6 (one of ‘Golden Delicious’) on Chr. 14 could also be used for not only negative but also positive selection to obtain watercored apples in the future.

In a transcriptome analysis of Japanese pear, 39 genes were found to be related to the watercore trait and located on Chr. 3, 7, or 11 (ref. ^53^). A GWAS of Japanese pear showed the highest (although not significant) association on Chr. 10 (ref. ^40^). These candidate regions of Japanese pear are different from this study, suggesting various mechanisms underlying the watercore development in pome fruits despite the high level of collinearity between the chromosomes of apples and pears^54^.

In the GWAS for WatCore, two and five genes, which were annotated as malate synthases and β-glucosidases, respectively, were found in the significant region on Chr. 14, whereas one gene, which was annotated as an ethylene receptor, was found in the significant region on Chr. 2. Sour perception has been reported to be enhanced in non-watercored apples^52^. Malate synthase is involved in the synthesis of malate, a precursor of malic acid. Malic acid is the main organic acid in mature apple fruit^55^. High expression of a putative malate synthase gene was observed in young, malate-accumulating grapes^56^. The flesh firmness of apple fruit has been reported to increase with enhancement of the watercore^52^. A reduction in firmness causes fruit softening. Fruit softening is generally caused by the modification of the cell wall, consisting of complex networks of polysaccharides, including cellulose^57^. In banana, the expression of several cellulose degradation-related enzymes, including β-glucosidase, was significantly upregulated during fruit softening^58^. The results suggested that the upregulated expression of β-glucosidase possibly facilitated cellulose degradation^58^. Ethylene is also considered to be involved in fruit softening in apples^57^. Ethylene receptors have been reported to act as negative regulators of ethylene response^59^. Watercored apples are accompanied by changes in multiple texture traits, such as sour perception and flesh firmness. Further investigation is needed to understand whether these changes are the cause or the result of the watercore.

In conclusion, the assignment of founder haplotypes to breeding populations improved the accuracy of GP for 7 traits and resolution of GWAS for 13 traits of 27 traits evaluated in this study. These results suggest that the information of founder haplotypes has good potential for genetic improvement of fruit quality traits in apples. The significant propagation of the founder haplotype with the largest genetic effect will help to understand the selection history of the founder haplotypes in the breeding program of Japanese apple varieties. Although we focused on the founder haplotypes in this study, the accuracy of GP may be further improved if the optimum number and length of the haplotypes are carefully selected for each trait. GS combined with superior haplotypes (Haplo-GS)^60^ could be a promising approach for fruit quality traits controlled by a large number of genes/haplotypes. In addition, the combination of GWAS based on founder haplotypes and SNP genotypes will be valuable for detecting candidate associations that cannot be detected with either of these methods.

## Materials and methods

### Plant materials

In this study, we used 185 cultivars and breeding lines of apple (*Malus* × *domestica* Borkh.), called the parental population (Supplementary Table S1), of which 21 lines were only used for genotyping and haplotyping with pedigree information. We also used 16 full-sib families consisting of 659 F_1_ individuals, called breeding populations (Supplementary Table S2). The breeding populations were derived from crosses among the 17 parental cultivars and breeding lines included in the parental population. These plant materials were grafted to popular rootstocks (JM1, JM7, M26, and so on) and cultivated for at least 4 years for phenotypic evaluation. The 95 seedlings in the juvenile phase derived from the cross ‘Fuji’ × ‘Golden Delicious’ (GD) were used for the construction of an integrated genetic map to confirm the loci of SNPs (Supplementary Data). All materials mentioned above were cultivated at the Apple Research Station, Institute of Fruit Tree Science, NARO.

### Evaluation of fruit quality traits

Detailed information for the phenotypic evaluation of 27 traits is presented in Table 2. The traits of the parental and breeding populations were evaluated from 1990 to 2015, and all traits except for MaxMeal were evaluated for 2–25 years. Two to five fruits, on average, per variety were used for the evaluation. Individual fruits were separately evaluated in terms of Weight, firmness (Firm), and degree of mealiness (DegMeal), and the average value was used as phenotypic data. Other traits were evaluated by the representative score/value of pooled samples or juice. The trait data evaluated over multiple years was adjusted considering the effect of the year using a mixed linear model implemented in the R package lme4 as described in Moriya et al.^13^. The best linear unbiased prediction (BLUP) of the genotype effect was used as the phenotypic value of a variety in subsequent GP and GWAS. The original phenotype of MaxMeal evaluated in 1 year strongly correlated (*r* = 0.91) with the BLUP of the related trait DegMeal, evaluated over multiple years.

### SNP genotyping, LD, and population structure

The genomic DNA of parental, breeding, and mapping populations were obtained from the leaves using a genomic-tip 20/G kit, DNeasy Plant Mini Kit (Qiagen, Hilden, Germany), or the CTAB method was implemented using the automated device PI-50α (Kurabo, Osaka, Japan). All samples were determined for their genotypes of 18,019 SNPs, as reported by Bianco et al.^5^, using the Infinium assay kit (Illumina, San Diego, CA, USA), according to the manufacturer’s instructions. The SNPs, which showed unclear cluster separation, or no polymorphism among tested samples, were removed from the analysis through visual inspection. Finally, 11,786 SNPs were obtained for the parental and breeding populations (Supplementary Data). The rate of missing SNP genotypes was 0.001. The genetic integrated map from ‘Fuji’ × ‘GD’ was constructed using JoinMap 4.1 (Kyazma, Wageningen, Netherlands), and the loci and order of each SNP were roughly determined. More detailed marker order or the loci of SNPs that were not mapped was estimated by the alignment of the SNP probe sequences to the reference genomes, ‘GD’ v1.0p^2^, and ‘GD’ doubled-haploid line (GDDH13) v1.1^61^. Some loci were inverted to decrease the discrepancies in the manual haplotype block described below.

LD patterns and population structure are key factors influencing the power of GWAS^18^ and the accuracy of GP^43,44^; therefore, we examined these features in the parental and breeding populations used in this study. The squared correlation coefficients (*r*^2^) between pairs of 11,144 SNPs, which had information of physical position in the GDDH13 v1.1^61^ genome, were calculated and plotted against physical distance between the corresponding markers in Mb. Local polynomial regression with kernel weight was conducted as described by Minamikawa et al.^40^ to model the relationship between the *r*^2^ values and physical map distances. The significance threshold of the *r*^2^ value was chosen based on the 95 percentile of the distribution of *r*^2^ values between pairs of unlinked markers. The genetic structures of the parental and combined parental and breeding populations were estimated using PCA, hierarchical clustering, and ADMIXTURE clustering, as described in Minamikawa et al.^40^.

### Estimation and tracing of founder haplotypes forward to populations

Fourteen founder haplotypes were estimated and traced automatically forward to a total of 844 individuals of the combined populations, according to the following four steps (Fig. 1). The first step was phasing and imputation of sporadic missing genotypes of the individuals. Four software algorithms, Beagle ver. 4.0^62^, findhap.f90 ver. 3^63^, MaCH ver. 1.0.16^64^, and fastPHASE ver. 1.4.8^65^, were used. Beagle and findhap.f90 use both LD and pedigree information, whereas MaCH and fastPHASE use only LD information. The second step was the estimation of the parental phases of the individuals. For each chromosome, one of the two phases with a higher correlation with that of the female (or male) parent than the male (or female) parent was estimated to be inherited from the female (or male) parent. The third step was the estimation of haplotype blocks inherited from the female (or male) parent based on the Viterbi algorithm, which is used to find an optimal path of hidden states, called the Viterbi path, in a hidden Markov model. We assumed that two phases of the female (or male) were two paths and proceeded with one of the two paths, adding penalty if the SNP scores in the path differed from that of the child (penalty = 1) or if we crossed over the path (i.e., recombination; penalty = 10). As a result, the path with the lowest penalty was chosen as the optimal path (i.e., haplotype blocks). The final step was to assign the 14 founder haplotypes to the haplotype blocks of the parental and breeding populations based on pedigree information. The region where we could not estimate whether it was inherited from the female or male parents kept the bi-allelic original SNP scores (15 or 16) in this study. Manual traces of the founder haplotypes were also performed based on the physical and linkage maps as described by Kunihisa et al.^31^. The automatic method was compared with the manual method, whose assignment of the haplotypes was assumed as correct, to evaluate the accuracy of the automatic method with four different phasing programs.

### Regression models for GP and GWAS

We used expectation–maximization (EM)-based BayesB^66^ and Markov chain Monte Carlo (MCMC)-based BayesB^67^ programs for GP and GWAS, respectively. The EM-based BayesB program was slightly modified to calculate the effect of the founder haplotypes in each marker. The MCMC-based BayesB program was also slightly modified to include a random effect with a covariance structure that depends on a genomic relational matrix into the model. The MCMC has also been successfully used in Bayesian modeling for GP^15^ and GWAS^68^. In general, the EM algorithm has been known to converge to local maxima or saddle points of posterior distributions, but the MCMC method has basically no such drawbacks when the MCMC calculation has sufficient mixing property. The MCMC method, however, is time-consuming and therefore might not be applied to cross-validations where multiple models need to be constructed with each training data for evaluating predictive ability. For this reason, we used the EM-based BayesB model for GP of each family of the breeding population. The hyperparameters of the model were set as *γ* = 0.01, *v* = 1, and *S* = 0.01, and minor allele frequency (MAF) = 0.01 (SNP) or 0.0001 (founder haplotype). We used founder haplotypes estimated automatically or manually or genotypes of each SNP as input variables of the model. To validate the accuracy of GP, we left one family out from the data, trained the model, and validated the accuracy of GP with the left-out family. This leave-one-family-out cross-validation was repeated for all 16 families. The aim of cross-validation is to confirm the accuracy of GP in a practical breeding program in which the phenotypic information of a family targeted by GS is generally not available. The prediction accuracy was evaluated using Pearson’s correlation coefficient (*r*) between observed and predicted genotypic values after combining the GP of all 16 families. When the estimated *r* was < 0, it was regarded as 0. The correlation was assessed with the test of no correlation, implemented in the “cor.test” function of R version 4.0.3^69^, when *r* was >0. The GP computation was performed on an Intel Xeon CPU E5-2640 v2 (2.00 GHz CPU 16-core) with 56 GB of RAM. To complete all the leave-one-family-out cross-validations of the 27 traits, 135 and 48 h were needed for founder haplotypes and SNPs, respectively.

The MCMC-based BayesB model with a genomic relationship matrix as a random effect was used for GWAS in the combined population to avoid spurious associations caused by the genetic stratification of the population. The genomic relationship matrix was computed using the “A.mat” function of the R package rrBLUP ver. 4.3^70^. The hyperparameters of the model were set as *v*_*β*_ = 4, $S_\beta ^2$ = 0.004 *v*_*e*_ = −2, $S_e^2$ = 0, *π* = 0.002 (*λ* = 20). MCMC cycles were repeated 50,000 times with the first 20,000 cycles used as burn-in. The number of MCMC cycles was determined based on Iwata et al.^71^. We used the founder haplotypes estimated from the Beagle-phasing data and SNPs for model construction. To identify significant markers, permutation analysis was conducted 100 times as described by Iwata et al.^67^, and then we chose the significance threshold at the 99 percentile of the value of a gamma parameter estimated for randomly permuted data. The contribution of the significant marker locus detected by GWAS with founder haplotypes on the phenotypic variance of the trait was calculated based on Eq. 1 in Iwata et al.^67^ (Supplementary Methods). Annotations of the genes found around the significant region were obtained from the Genome Database for Rosaceae (GDR) (https://www.rosaceae.org)^72^ and Phytozome version 12.1.6 (https://phytozome.jgi.doe.gov/pz/portal.html). The LD heatmaps of the regions surrounding significant associations on Chr. 1 and Chr. 9 for PerOC and Chr. 2 and Chr. 14 for WatCore were constructed using the R package LDheatmap ver. 1.0–4^73^. The GWAS computation was performed on an Intel Xeon CPU E5-2687W v4 (3.00 GHz dual CPU 24-core) with 128 GB of RAM. To complete all GWAS of the 27 traits, 150 and 8 h were needed for founder haplotypes and SNPs, respectively.

To examine whether the propagation of founder haplotypes, which had the largest and smallest genetic effects in GWAS, have a nonrandom pattern (e.g., significant increase or decrease in the proportion of a haplotype in the parental population compared to the initial frequency (i.e., 1 of the 14 founder haplotypes, 0.07)), a random transmission test of founder haplotypes based on the pedigree information was performed. One of the two inherited founder haplotypes was randomly selected based on the binomial distribution. This simulation scenario was repeated 10,000 times. Finally, the probability (*p* value) of more or less than the observed frequency of the founder haplotype in the frequency distribution of founder haplotypes generated from the simulation was calculated to test for significance. The propagation of the founder haplotype was visualized using the software Helium^74^.

## Supplementary information

Supplementary information

Supplementary data
