# Supplementary material for: Tracing founder haplotypes of Japanese apple varieties: application in genomic prediction and genome-wide association study
Source: Hortic Res. 2021 Mar 1;8:49. doi: 10.1038/s41438-021-00485-3 (PMC7917097; doi:10.1038/s41438-021-00485-3)
Supplement: Supplementary file 1 — Supplementary information [file 41438_2021_485_MOESM1_ESM.pdf]

Supplementary information

Tracing founder haplotypes of Japanese apple varieties: Application in genomic prediction and genome-wide association study

Mai F. Minamikawa[1], Miyuki Kunihisa[2], Koji Noshita[1], Shigeki Moriya[3], Kazuyuki Abe[3], Takeshi Hayashi[4], Yuichi Katayose[4], Toshimi Matsumoto[4,5], Chikako Nishitani[2], Shingo Terakami[2], Toshiya Yamamoto[2], Hiroyoshi Iwata[1]*

[1]Laboratory of Biometry and Bioinformatics, Department of Agricultural and Environmental Biology, Graduate School of Agricultural and Life Sciences, The University of Tokyo, 1-1-1 Yayoi, Bunkyo, Tokyo 113-8657, Japan

[2]Institute of Fruit Tree and Tea Science, National Agriculture and Food Research Organization (NARO), 2-1 Fujimoto, Tsukuba, Ibaraki 305-8605, Japan

[3]Division of Apple Research, Institute of Fruit Tree and Tea Science, NARO, 92-24 Shimokuriyagawa Nabeyashiki, Morioka, Iwate 020-0123, Japan

[4]Institute of Crop Science, NARO, 2-1-2 Kannondai, Tsukuba, Ibaraki 305-8518, Japan

[5]Institute of Agrobiological Sciences, NARO, 1-2 Owashi, Tsukuba, Ibaraki 305-8634, Japan

*To whom correspondence should be addressed: E-mail: hiroiwata@g.ecc.u-tokyo.ac.jp

Supplementary Tables S1-S4
Supplementary Figures S1-S7
Supplementary Methods

Supplementary Table S1. Parental apple population used in this study.

Variety No.	Name	Parentage	Developer (Origin[e])
1	Aikanokaori	Fuji × Tsugaru[b]	H. Fujimaki
2	Aizunohoppe[a]	Fuji × Jonathan	FATC
3	Akagi	Golden Delicious × Jonathan	GATC
4	Akane	Jonathan × Worcester Pearmain	NIFTS
5	Akibae	Sensyu × Tsugaru	T. Odagiri
6	Akita Gold	Golden Delicious × Fuji	AFTES
7	Akitabeniakari	Orin × Sensyu	AFTES
8	Akitabenihoppe[a]	Sensyu × Sansa	AFTES
9	American Summer Pearmain	? × ?	- (USA)
10	Aori 11	Kita no Sachi × Jersey Mac	AITC
11	Aori 12	Kita no Sachi × Jersey Mac	AITC
12	Aori 13	Richared Delicious[c] × Tsugaru	AITC
13	Aori 15	Fuji × Aori 3	AITC
14	Aori 16	(Akane × Rero11) × Natsumidori (Kitakami × Meku10 (Tsugaru × American Summer Pearmain))	AITC
15	Aori 21	Fuji × Rei8 (Toko × Jonathan)	AITC
16	Aori 24[a]	Granny Smith × Rei8 (Toko × Jonathan)	AITC
17	Aori 25[a]	Merrow × Liberty	AITC
18	Aori 27	Kinsei × Mahe7	AITC
19	Aori 29[a]	Tsugaru × Sansa	AITC
20	Aori 3	Toko × Richared Delicious[c]	AITC
21	Beniminori	Tsugaru × Gala	NIFTS
22	Beninomai	Tsugaru[b] × ?	Tenkoen. Co. Ltd
23	Beniroman	Shinano Red × Sansa	T. Takano
24	Binikohaku[a]	Hozuri × Yoko	FATC
25	Braeburn	Lady Hamilton × ?	O. Moran (NZ)
26	Catarina	Fuji × ?	- (Brasil)
27	Chinatsu	Akane × Stark Earliest	NIFTS
28	Cox's Orange Pippin	? × ?	R. Cox (UK)
29	Cute	Sensyu × Tsugaru	Ishidou. Co. Ltd
30	Delicious	Winesap × ?	J. Hiatt (USA)
31	Elstar	Golden Delicious × ?	- (Netherland)
32	First Lady	Sansa × Tsugaru	YPHES
33	Fuji	Ralls Janet × Delicious	NIFTS
34	Fujiwararoman	Shinano Gold × Fuji	T. Takano
35	Gala	Kidd's Orange Red × Golden Delicious	W. McKenzie (NZ)
36	Golden Delicious	Grimes Golden × ?	A. Mullins (USA)
37	Golden Melon	Golden Delicious × Indo	AITC
38	Goldroman	Shinano Gold × Tsugaru	T. Takano
39	GoldRush	Golden Delicious × Coop17	PRI (USA)
40	Granny Smith	? × ?	M.A. Smith (Australia)
41	Gunma Meigetu	Akagi × Fuji	GATC
42	HAC6[a]	Golden Delicious × (McIntosh × Red Delicious[c])	HRO
43	Haruka	Golden Delicious × Delicious	K. Yokota
44	Hatsuaki	Jonathan × Golden Delicious	NIFTS
45	Hida	Fuji × Golden Delicious[b]	T. Sunabara
46	Himekami	Fuji × Jonathan	NIFTS
47	Hinoazuma	Sensyu × Himekami	FATC
48	Honey Queen	Megumi × Sekaiichi	GATC
49	Honeygold	Golden Delicious × Haralson	MHRC (USA)
50	Hoozuri	Fuji × Jonathan	FATC
51	Indo	? × ?	-
52	Iwakami	Fuji × Jonathan	NIFTS
53	Iwaki	Tsugaru[b] × Jonathan	AITC
54	Iwate 7[a]	Tsugaru × Pricilla	IARC
55	Jersey Mac[a]	? × ?	NJAES (USA)
56	Jonathan	? × ?	- (USA)
57	Kanki	Sensyu × Tsugaru	S. Kudo
58	Kaori (Romu50)	Richared Delicious[c] × ?	AITC
59	Kidd's Orange Red	Cox's Orange Pippin × Delicious	J. H. Kidd (NZ)
60	Kinsei	Golden Delicious × Delicious strain	H. Sato

Supplementary Table S1. (*Continued*)

Variety No.	Name	Parentage	Developer (Origin[e])
61	Kinshu	Sensyu × 4-4349	NIFTS
62	Kio	Orin × Sensyu	IARC
63	Kita no Sachi[a]	Tsugaru × American Summer Pearmain	AITC
64	Kitakami	Tohoku 2 × Redgold	NIFTS
65	Kitaro	Fuji × Hatsuaki	NIFTS
66	Kizashi	Gala × Stark Earliest	NIFTS
67	Koko	(Starking Delicious[c] × Golden Delicious) × Fuji	Y. Shiozaki
68	Kotaro	Fuji × Hatsuaki	NIFTS
69	Mahe7	(Indo × Golden Delicious) × Redgold	AITC
70	Maoi	Mantet × HAC6	HRO
71	McIntosh	? × ?	J. McIntosh (Canada)
72	Megumi	Ralls Janet × Jonathan	AITC
73	Mellow	(Golden Delicious × Indo) × Golden Delicious	AITC
74	Merton Worcester	Cox's Orange Pippin × ?	John Innes Institute (UK)
75	Miki Life	Sensyu × Tsugaru	S. Kudo
76	Morinokagayaki	Tsugaru × Gala	NIFTS
77	Morioka 44	Gala × Akane	NIFTS
78	Morioka 45	Gala × Akane	NIFTS
79	Morioka 46	Gala × ?	NIFTS
80	Morioka 47[a]	Gala × Akane	NIFTS
81	Morioka 48	Kitakami × Stark Earliest	NIFTS
82	Morioka 51	Hatsuaki × Golden Delicious	NIFTS
83	Morioka 53	Hatsuaki × Fuji	NIFTS
84	Morioka 54	Hatsuaki × Fuji	NIFTS
85	Morioka 57	Jonathan × Hatsuaki	NIFTS
86	Morioka 58	Hatsuaki × Fuji	NIFTS
87	Morioka 59	Kitakami × Hatsuaki	NIFTS
88	Morioka 60	Hatsuaki × Starking Delicious[c]	NIFTS
89	Morioka 61	Tsugaru × Kitakami	NIFTS
90	Morioka 62	Akane × Maigold	NIFTS
91	Morioka 64	Iwaki × Sansa	NIFTS
92	Morioka 65	Orin × Sansa	NIFTS
93	Morioka 66	Sansa × 4-4349	NIFTS
94	Morioka 68	Sansa × Sensyu	NIFTS
95	Morioka 69	Sansa × Sekaiichi	NIFTS
96	Morioka 71	Fuji × Tsugaru	NIFTS
97	Na-130	McIntosh × Worcester Pearmain	NIFTS
98	Na-56[a]	? × ?	NIFTS
99	Narihoko	Golden Delicious × Fuji	T. Narita
100	Nero26	(Jonathan × Golden Delicious) × Richared Delicious[c]	AITC
101	Orei	Golden Delicious × Delicious	AITC
102	Orin	Golden Delicious × Indo	T. Otsuki
103	Ouka	Tsugaru × Priscilla	IARC
104	Ozenokurenai	Morioka 47 × Morioka 46	GATC, NIFTS
105	Priscilla	Delicious × PRI610-2	PRI (USA)
106	Ralls Janet	? × ?	- (USA)
107	RD122[a]	Richared Delicious[c] × ?	-
108	Redgold	Golden Delicious × Richared Delicious[c]	F. A. Schell (USA)
109	Rero11	Toko × Richared Delicious[c]	AITC
110	Ro-125	Ralls Janet × Delicious	NIFTS
111	Ro-329	Ralls Janet × Delicious	NIFTS
112	Rose Pearl	Fuji × Pink Pearl	NIFTS
113	Rubinnette	Golden Delicious × Cox's Orange Pippin	W. Hauenstein (Switzerlanc
114	Ruby Sweet	apple genetic resource (JP114069) × Fuji	NIFTS
115	Sansa	Akane × Gala	NIFTS
116	Scarlett O'Hara™[a]	PCF 2-134 × PRI 669-205	PRI (USA)
117	Seirin	Redgold × Fuji	H. Oyamada *et al*.
118	Sekaiichi	Delicious × Golden Delicious	AITC
119	Sensyu	Toko × Fuji	AFTES
120	Shinano Dolce	Golden Delicious × Sensyu	NFTES

Supplementary Table S1. (*Continued*)

Variety No.	Name	Parentage	Developer (Origin[e])
121	Shinano Gold	Golden Delicious × Sensyu	NFTES
122	Shinano Hoppe	Akane × Fuji	NFTES
123	Shinano Piccoro	Golden Delicious × Akane	NFTES
124	Shinano Pucchi	Tsugaru × Sansa	NFTES
125	Shinano Red	Tsugaru × Vista Bella	NFTES
126	Shinano Sweet	Fuji × Tsugaru	NFTES
127	Silken	Honeygold × Sunrise	PARC (Canada)
128	Sinsekai	Fuji × Akagi	GATC
129	Slim Red	Fuji × Akagi	GATC
130	Sour Rouge[a]	Fuji × Jonathan[b]	MIAH
131	Stark Earliest	? × ?	D. Bonner (USA)
132	Sunrise[a]	McIntosh × Golden Delicious	-
133	Syusei	Fuji × Tsugaru	IAFRC
134	Telamon	Wijcik[d] × Golden Delicious	EMRS (UK)
135	Tohoku2	Worcester Pearmain × McIntosh	NIFTS
136	Toki	Orin × Fuji	Harada Shubyo Co. Ltd.
137	Toko	Golden Delicious × Indo	AITC
138	Trajan	Golden Delicious × Wijcik[d]	EMRS (UK)
139	Tsugaru	Golden Delicious × Jonathan	AITC
140	Tuscan	Wijcik[d] × Greensleeves	EMRS (UK)
141	Worcester Pearmain	? × ?	Mr. Hale (UK)
142	Yoko	Golden Delicious × Jonathan	GATC
143	230-1[a]	Tsugaru × Sansa	AITC
144	4-161	Hatsuaki × Starking Delicious[c]	NIFTS
145	4-23[a]	Fuji × Mahe7	AITC
146	4-4349	Tsugaru × Iwakami	NIFTS
147	4-547	Fuji × Hatsuaki	NIFTS
148	5-12786	Fuji × 8H-2-26	NIFTS
149	5-13097[a]	Sansa × 8H-2-26	NIFTS
150	5-3430	Sensyu × Sansa	NIFTS
151	5-3645	Sansa × Tsugaru	NIFTS
152	5-5102	Tsugaru × Fuji	NIFTS
153	5-5471	Sansa × Tsugaru	NIFTS
154	5-6393	Akane × 4-23	NIFTS
155	5-6716	Tsugaru × Kizashi	NIFTS
156	5-7529	Sansa × Morioka 48	NIFTS
157	5-7572	Sansa × Kinsei	NIFTS
158	6-2498	Sansa × Sekaiichi	NIFTS
159	6-5860[a]	Sansa × 8H-2-26	NIFTS
160	6-6832	Sansa × Morioka 48	NIFTS
161	6-7621	Sansa × Scarlett O'Hara™	NIFTS
162	6-7729	Sansa × Coop29	NIFTS
163	6-7810	Sensyu × Scarlett O'Hara™	NIFTS
164	6-8083	Fuji × Coop29	NIFTS
165	6-8084	Fuji × Coop29	NIFTS
166	6-8098	Fuji × Coop29	NIFTS
167	6-8491	Chinatsu × ?	NIFTS
168	6-8507	Chinatsu × Morioka 48	NIFTS
169	6-8572	Akane × Shinano Sweet	NIFTS
170	6-8584	Akane × Shinano Sweet	NIFTS
171	6-8585	Akane × Shinano Sweet	NIFTS
172	6-8608	Akane × Shinano Sweet	NIFTS
173	6-9191[a]	Kotaro × ?	NIFTS
174	6-9706	Chinatsu × 5-6716	NIFTS
175	7-1192	Gala × Morioka 64	NIFTS
176	7-152	Sensyu × Morioka 64	NIFTS
177	7-2170	Sansa × 5-12786	NIFTS
178	7-2241	Sansa × 5-12786	NIFTS
179	7-2286	Sansa × 5-12786	NIFTS
180	7-3111	Beniminori × Chinatsu	NIFTS
181	7-3635	Shinano Gold × Morioka 64	NIFTS
182	7-3762	Morioka 61 × Silken	NIFTS
183	7-3888	Silken × Kotaro	NIFTS
184	7-4787	Kotaro × Haruka	NIFTS
185	8H-2-26	(Wijcik[d] × Golden Delicious) × (Wijcik[d] × Golden Delicious)	- (Canada)

[a] Varieties were used only for haplotype analysis. Phenotypes of the varieties were not evaluated in this study.

[b] Candidate paternal parents predicted in this study, with SNP genotypes.

[c] 'Richared Delicious', 'Red Delicious' and 'Starking Delicious' are sports of 'Delicious'.

[d] 'Wijcik' is a sport of 'McIntosh'.

[e] The origins except Japan were shown.

AFTES: Akita Fruit-Tree Experiment Station, AITC: Aomori Prefectural Industrial Technology Research Center, EMRS: East Malling Research Station, FATC: Fukushima Agricultural Technology Center, GATC: Gunma Agricultural Technology Center, HRO: Hokkaido Reserch Organization, IAFRC: Ishikawa Agriculture and Forestry Research Center, IARC: Iwate Agricultural Research Center, MIAH: Miyagi prefectural Institute of Agriculture and Horticulture, MHRC: Minnesota Horticultural Research Center, NFTES: Nagano Fruit Tree Experiment Station, NIFTS: NARO Institute of Fruit Tree Science, NJAES: New Jersey Agricultural Experiment Station, PARC: Pacific Agri-Food Research Centre, PRI: Purdue, Rutgers, Illinois Co-op (USA), YPHES: Yamagata Prefectural Horticultural Experiment Station

Supplementary Table S2. Breeding apple population used in this study.

Family No.	Parentage	Number of genotypes
1	Fuji × Sansa	20
2	Hatsuaki × Megumi	57
3	Orin × Akane	131
4	Shinano Gold × Morioka 64	59
5	Sinsekai × Morinokagayaki	60
6	Fuji × Hatsuaki	50
7	Fuji × Tsugaru	30
8	Tsugaru × Delicious	37
9	Kitaro × Akane	50
10	Sansa × Jonathan	20
11	Sensyu × Kotaro	28
12	Orin × Sansa	20
13	Sensyu × Sansa	18
14	Jonathan × Delicious	19
15	Sansa × Tsugaru	30
16	Kotaro × Kinshu	30

Supplementary Table S3. Significant loci detected by GWAS based on founder haplotypes (Fig. 3).
The top 20 loci were listed for traits in which more than 20 significant loci were detected.

Trait	Marker name	Chromosome	Order	GD Genome v1.0		GDDH13 Genome v1.1		Posterior prob. of QTL (γ)	Founder haplotypes with the largest or smallest genetic effects: Positive (the largest effect)				Negative (the smallest effect)				Contribution rate
				Chromosome	Position (bp)	Chromosome	Position (bp)		Founder haplotype	Founder	genetic effect (β)	Frequency in parental population	Founder haplotype	Founder	genetic effect (β)	Frequency in parental population	
PickDay	SNP_FB_1108208	1	103	NA	NA	Chr01	12989414	0.072	16	NA	0.184	NA	1	Ralls Janet	-0.174	0.132**	0.00004
PickDay	RosBREEDSNP_SNP_AG_31160644_Lg3_01779_MAF30_535055_exon1	3	1864	chr3	31160645	Chr03	30411691	0.936	2	Ralls Janet	9.276	0.068	9	Worcester Pearmain	-8.527	0.035	0.07764
PickDay	SNP_FB_0546498	3	1874	chr3	32033159	Chr03	31330141	0.072	2	Ralls Janet	0.705	0.068	9	Worcester Pearmain	-0.688	0.038	0.00043
PickDay	GDsnp01651	5	2873	chr5	10324165	NA	NA	0.539	16	NA	3.448	NA	15	NA	-2.648	NA	0.00945
PickDay	SNP_FB_1052855	10	6220	NA	NA	Chr10	14877221	0.234	2	Ralls Janet	0.731	0.008	16	NA	-1.353	NA	0.00176
PickDay	SNP_FB_1052863	10	6223	NA	NA	Chr10	14878209	0.263	2	Ralls Janet	0.709	0.008	15	NA	-1.579	NA	0.00250
PickDay	RosBREEDSNP_SNP_AG_32730568_Lg10_RosCOS3231_MAF50_492103_exon1	10	6586	chr10	32730569	Chr10	38213067	0.114	2	Ralls Janet	0.606	0.008	1	Ralls Janet	-0.385	0.081	0.00023
PickDay	RosBREEDSNP_SNP_CT_32751652_Lg10_RosCOS3231_MAF40_MDP0000317596_exon2	10	6588	chr10	32751653	Chr10	38237118	0.063	2	Ralls Janet	0.257	0.008	1	Ralls Janet	-0.184	0.081	0.00006
PickDay	SNP_FB_0045154	10	6590	chr10	32754019	Chr10	38239484	0.067	2	Ralls Janet	0.332	0.008	1	Ralls Janet	-0.202	0.081	0.00008
PickDay	SNP_FB_0045914	10	6592	chr10	33223382	Chr10	38694535	0.074	2	Ralls Janet	0.352	0.008	9	Worcester Pearmain	-0.236	0.057	0.00009
PickDay	SNP_FB_0063313	11	6842	chr11	7566331	NA	NA	0.093	15	NA	0.525	NA	16	NA	-0.603	NA	0.00019
PickDay	RosBREEDSNP_SNP_TC_32489536_Lg12_00362_MAF40_151627_exon1	12	8011	chr12	32489536	Chr12	28930199	0.355	16	NA	2.599	NA	15	NA	-2.691	NA	0.00350
PickDay	RosBREEDSNP_SNP_TG_33117888_Lg12_135549_MAF20_135549_exon1	12	8029	chr12	33117888	Chr12	29543519	0.124	16	NA	1.327	NA	15	NA	-0.908	NA	0.00045
PickDay	RosBREEDSNP_SNP_TC_33687206_Lg12_02008_MAF40_287310_exon1	12	8058	chr12	33687206	Chr12	30196011	0.300	16	NA	2.375	NA	15	NA	-2.526	NA	0.00271
PickDay	GDsnp02844	15	10031	chr15	2514751	Chr15	29698160	0.062	6	Golden Delicious	0.228	0.178	7	Jonathan	-0.193	0.051	0.00010
PickDay	SNP_FB_0785569	15	10033	chr9	8859330	Chr15	30253581	0.117	6	Golden Delicious	0.486	0.178	15	NA	-0.512	NA	0.00039
PickDay	SNP_FB_0304198	15	10037	chr15	30339216	Chr15	31116111	0.515	6	Golden Delicious	2.221	0.178	15	NA	-2.508	NA	0.00841
PickDay	SNP_FB_0977426	15	10055	chr4	391585	Chr15	31646987	0.099	16	NA	0.551	NA	15	NA	-0.438	NA	0.00029
PickDay	SNP_FB_1075562	16	10757	NA	NA	Chr16	8848914	0.596	12	Indo	4.814	0.008	16	NA	-3.375	NA	0.01674
PickDay	GDsnp02194	16	10769	chr16	6803057	Chr16	9084376	0.404	12	Indo	3.511	0.008	15	NA	-2.743	NA	0.00878
OvColor	SNP_FB_0817523	9	5885	chr9	32674143	Chr09	33741199	1.000	16	NA	0.424	NA	4	Delicious	-0.558	0.076	0.20912
OvColor	SNP_FB_1030164	14	9049	NA	NA	Chr14	11060063	0.061	7	Jonathan	0.009	0.073	15	NA	-0.009	NA	0.00006
OvColor	SNP_FB_1030165	14	9050	NA	NA	Chr14	11060150	0.045	15	NA	0.006	NA	5	Golden Delicious	-0.006	0.105	0.00003
OvColor	SNP_FB_1030185	14	9053	NA	NA	Chr14	11062403	0.031	7	Jonathan	0.004	0.073	15	NA	-0.006	NA	0.00002
OvColor	GDsnp02426	14	9066	chr14	18418692	Chr14	15595330	0.145	7	Jonathan	0.021	0.073	15	NA	-0.028	NA	0.00046
OvColor	SNP_FB_0239463	14	9079	chr14	20343500	Chr14	14952814	0.032	7	Jonathan	0.005	0.103	15	NA	-0.004	NA	0.00001
PerOC	RosBREEDSNP_SNP_GA_29128835_Lg1_10005_MAF10_326880_exon1	1	426	chr1	29129846	Chr01	27745994	0.037	6	Golden Delicious	0.008	0.124	1	Ralls Janet	-0.009	0.005	0.00002
PerOC	SNP_FB_0439059	1	427	chr1	29153599	Chr01	27728004	0.030	6	Golden Delicious	0.006	0.124	1	Ralls Janet	-0.004	0.005	0.00001
PerOC	SNP_FB_0439060	1	428	chr1	29153851	Chr01	27727752	0.065	6	Golden Delicious	0.016	0.124	1	Ralls Janet	-0.014	0.005	0.00010
PerOC	SNP_FB_0439066	1	430	chr1	29154455	Chr01	27727148	0.034	6	Golden Delicious	0.007	0.124	5	Golden Delicious	-0.005	0.057*	0.00002
PerOC	SNP_FB_0439071	1	431	chr1	29154820	Chr01	27726783	0.030	6	Golden Delicious	0.006	0.124	1	Ralls Janet	-0.007	0.005	0.00001
PerOC	RosBREEDSNP_SNP_CA_29158464_Lg1_10005_MAF40_519911_exon1	1	435	chr1	29158464	Chr01	27723149	0.037	6	Golden Delicious	0.008	0.124	5	Golden Delicious	-0.007	0.057*	0.00002
PerOC	RosBREEDSNP_SNP_CT_31701102_Lg9_00206_MAF50_1673498_exon1	9	5843	chr9	31701104	Chr09	32680402	0.031	1	Ralls Janet	0.017	0.114*	12	Indo	-0.019	0.008	0.00014
PerOC	SNP_FB_0816029	9	5851	chr9	31858548	Chr09	32859498	0.073	1	Ralls Janet	0.038	0.114*	12	Indo	-0.039	0.008	0.00089
PerOC	SNP_FB_0816821	9	5867	chr9	32298587	Chr09	33373473	0.036	1	Ralls Janet	0.021	0.111*	12	Indo	-0.021	0.008	0.00020
PerOC	RosBREEDSNP_SNP_TC_32540237_Lg9_01066_MAF50_787255_exon1	9	5873	chr9	32540239	Chr09	33587227	0.110	1	Ralls Janet	0.062	0.111*	12	Indo	-0.074	0.008	0.00219
PerOC	SNP_FB_0817506	9	5878	chr9	32671269	Chr09	33738325	0.077	10	Worcester Pearmain	0.045	0.084*	12	Indo	-0.046	0.008	0.00096
PerOC	SNP_FB_0817519	9	5884	chr9	32673314	Chr09	33740370	0.041	10	Worcester Pearmain	0.025	0.084*	12	Indo	-0.026	0.008	0.00026
PerOC	SNP_FB_0817523	9	5885	chr9	32674143	Chr09	33741199	0.077	10	Worcester Pearmain	0.043	0.084*	12	Indo	-0.049	0.008	0.00102
PerOC	RosBREEDSNP_SNP_GA_32711352_Lg9_01416_MAF50_450241_exon1	9	5888	chr9	32711354	Chr09	33754289	0.155	10	Worcester Pearmain	0.087	0.084*	12	Indo	-0.096	0.008	0.00435
PerOC	RosBREEDSNP_SNP_AG_33217423_Lg9_00332_MAF20_390087_exon1	9	5903	chr9	33220611	Chr09	34332642	0.038	10	Worcester Pearmain	0.020	0.084*	12	Indo	-0.024	0.008	0.00023
PerOC	RosBREEDSNP_SNP_GA_33227831_Lg9_00332_MAF40_MDP0000175442_exon2	9	5904	chr9	33214351	Chr09	34343047	0.052	10	Worcester Pearmain	0.028	0.084*	12	Indo	-0.031	0.008	0.00042
PerOC	RosBREEDSNP_SNP_TC_33439813_Lg9_129159__129159_exon2	9	5906	chr9	33439815	Chr09	34549325	0.039	10	Worcester Pearmain	0.024	0.084*	12	Indo	-0.027	0.008	0.00024
PerOC	SNP_FB_0820931	9	5923	chr9	34540454	Chr09	36358250	0.058	10	Worcester Pearmain	0.031	0.084*	12	Indo	-0.028	0.008	0.00049
PerOC	SNP_FB_0820945	9	5926	chr9	34544978	Chr09	36362808	0.064	10	Worcester Pearmain	0.034	0.084*	12	Indo	-0.034	0.008	0.00062
PerOC	SNP_FB_0820949	9	5928	chr9	34545889	NA	NA	0.046	10	Worcester Pearmain	0.024	0.084*	12	Indo	-0.023	0.008	0.00031

Supplementary Table S3. ***(Continued)***

Trait	Marker name	Chromosome	Order	GD Genome v1.0		GDDH13 Genome v1.1		Posterior prob. of QTL (γ)	Founder haplotypes with the largest or smallest genetic effects								Contribution rate
									Positive (the largest effect)				Negative (the smallest effect)				
				Chromosome	Position (bp)	Chromosome	Position (bp)		Founder haplotype	Founder	genetic effect (β)	Frequency in parental population	Founder haplotype	Founder	genetic effect (β)	Frequency in parental population	
RusTop	SNP_FB_0586209	4	2458	chr4	20123263	Chr04	27122709	0.031	14	Cox's Orange Pippin	0.007	0.008	2	Ralls Janet	-0.009	0.105	0.00005
RusTop	RosBREEDSNP_SNP_TC_20512923_Lg4_01773_MAF30_MDP0000205358_exon1	4	2470	chr4	20512466	Chr04	27632513	0.046	14	Cox's Orange Pippin	0.013	0.008	12	Indo	-0.014	0.016	0.00010
RusTop	RosBREEDSNP_SNP_AG_20969382_Lg4_00686_MAF20_1653787_exon1	4	2482	chr4	20969385	Chr04	28082797	0.144	14	Cox's Orange Pippin	0.050	0.008	12	Indo	-0.047	0.016	0.00131
RusTop	RosBREEDSNP_SNP_AG_21422131_Lg4_01558_MAF30_MDP0000270110_exon4	4	2499	chr4	21422134	Chr04	28478994	0.039	10	Worcester Pearmain	0.010	0.035	12	Indo	-0.012	0.016	0.00007
RusTop	SNP_FB_0588458	4	2502	chr4	21435063	Chr04	28491917	0.073	10	Worcester Pearmain	0.019	0.035	12	Indo	-0.028	0.016	0.00031
RusTop	GDsnp01558	4	2510	chr4	21460139	Chr04	28516576	0.039	10	Worcester Pearmain	0.010	0.035	12	Indo	-0.014	0.016	0.00008
RusTop	SNP_FB_0986140	4	2516	chr4	21953086	Chr04	28955234	0.054	10	Worcester Pearmain	0.011	0.038	12	Indo	-0.016	0.016	0.00014
RusTop	GDsnp01965	4	2518	chr4	21959295	Chr04	28961445	0.029	10	Worcester Pearmain	0.005	0.038	12	Indo	-0.007	0.022	0.00004
RusTop	SNP_FB_0589657	4	2520	chr4	22204633	NA	NA	0.028	10	Worcester Pearmain	0.006	0.038	12	Indo	-0.007	0.022	0.00004
RusTop	SNP_FB_0589665	4	2522	chr4	22206378	Chr04	29188637	0.130	10	Worcester Pearmain	0.030	0.038	12	Indo	-0.037	0.022	0.00094
RusTop	SNP_FB_0589674	4	2527	chr4	22211306	Chr04	29193703	0.209	10	Worcester Pearmain	0.049	0.038	12	Indo	-0.058	0.022	0.00244
RusTop	SNP_FB_0590700	4	2539	chr4	22736589	Chr04	29757980	0.033	10	Worcester Pearmain	0.007	0.038	12	Indo	-0.008	0.022	0.00005
RusTop	SNP_FB_0590704	4	2541	chr4	22738411	Chr04	29759802	0.030	10	Worcester Pearmain	0.005	0.038	12	Indo	-0.007	0.022	0.00004
RusBody	SNP_FB_0441428	1	455	chr1	30753466	Chr01	28685721	0.033	5	Golden Delicious	0.003	0.105	2	Ralls Janet	-0.003	0.149**	0.00001
RusCal	SNP_FB_0487035	2	1112	chr2	26915236	Chr02	26060054	0.263	8	Jonathan	0.057	0.108	10	Worcester Pearmain	-0.070	0.100**	0.00569
RusCal	SNP_FB_0490066	2	1143	chr2	30295908	Chr02	28380171	0.088	8	Jonathan	0.020	0.108	10	Worcester Pearmain	-0.023	0.100**	0.00063
RusCal	SNP_FB_0490085	2	1147	chr2	30304008	Chr02	28372071	0.361	8	Jonathan	0.083	0.108	10	Worcester Pearmain	-0.095	0.100**	0.01109
RusCal	RosBREEDSNP_SNP_GA_30314379_Lg2_01251_MAF50_MDP0000246555_exon1	2	1149	chr2	30314380	Chr02	28361642	0.212	8	Jonathan	0.048	0.108	10	Worcester Pearmain	-0.056	0.100**	0.00383
RusCal	SNP_FB_0490931	2	1156	chr2	30844883	Chr02	27961077	0.087	8	Jonathan	0.021	0.092	10	Worcester Pearmain	-0.021	0.100**	0.00061
RusCal	SNP_FB_0221133	14	8963	chr14	6802606	Chr14	4109938	0.031	8	Jonathan	0.004	0.095	1	Ralls Janet	-0.005	0.005	0.00001
RusCal	SNP_FB_0221135	14	8964	chr14	6803108	Chr14	4110478	0.032	8	Jonathan	0.004	0.095	1	Ralls Janet	-0.006	0.005	0.00002
RusCal	SNP_FB_0335238	16	10549	chr16	1191393	Chr16	3003678	0.073	11	Indo	0.015	0.011	6	Golden Delicious	-0.008	0.078	0.00017
RusCal	SNP_FB_0335240	16	10550	chr16	1191830	Chr16	3004115	0.031	11	Indo	0.006	0.011	3	Delicious	-0.003	0.165*	0.00003
RusCal	SNP_FB_0335241	16	10551	chr16	1191967	Chr16	3004252	0.025	11	Indo	0.004	0.011	6	Golden Delicious	-0.003	0.078	0.00001
RusCal	SNP_FB_0335320	16	10554	chr16	1216587	Chr16	3025410	0.027	11	Indo	0.005	0.011	8	Jonathan	-0.003	0.146*	0.00002
RusCal	RosBREEDSNP_SNP_AC_1452699_Lg16_MDP0000303483_MAF50_MDP0000303483_exon2	16	10573	chr16	1452699	Chr16	3304530	0.044	1	Ralls Janet	0.008	0.003**	8	Jonathan	-0.006	0.151*	0.00004
RusCal	SNP_FB_1074678	16	10574	NA	NA	Chr16	3362258	0.046	11	Indo	0.008	0.011	8	Jonathan	-0.006	0.151*	0.00005
RusCal	RosBREEDSNP_SNP_CT_1540624_Lg16_LAR1_MAF40_1618769_exon2	16	10578	chr16	1540624	Chr16	3409834	0.062	11	Indo	0.013	0.011	8	Jonathan	-0.009	0.151*	0.00011
RusCal	RosBREEDSNP_SNP_TC_1571002_Lg16_01734_MAF40_216397_exon1	16	10579	chr16	1571002	Chr16	3439690	0.029	11	Indo	0.006	0.011	8	Jonathan	-0.004	0.151*	0.00002
RusCal	SNP_FB_0336036	16	10581	NA	NA	Chr16	3479443	0.062	11	Indo	0.012	0.011	8	Jonathan	-0.008	0.151*	0.00010
RusCal	SNP_FB_0336046	16	10582	NA	NA	Chr16	3482544	0.029	11	Indo	0.005	0.011	8	Jonathan	-0.005	0.151*	0.00002
RusCal	SNP_FB_0336063	16	10583	chr16	1617578	Chr16	3486139	0.026	11	Indo	0.005	0.011	8	Jonathan	-0.003	0.151*	0.00002
RusCal	SNP_FB_1074699	16	10588	NA	NA	Chr16	3774681	0.027	11	Indo	0.004	0.011	3	Delicious	-0.003	0.165*	0.00002
RusCal	GDsnp00047	16	10590	chr16	1930716	Chr16	3856528	0.045	11	Indo	0.008	0.011	8	Jonathan	-0.006	0.154*	0.00005
Scarf	SNP_FB_0502408	2	1337	chr2	38327872	NA	NA	0.037	10	Worcester Pearmain	0.002	0.078*	8	Jonathan	-0.002	0.086	0.00003
Scarf	SNP_FB_0662268	6	3705	chr6	12791489	Chr06	18752930	0.037	7	Jonathan	0.003	0.041	2	Ralls Janet	-0.002	0.081	0.00004
Scarf	GDsnp02763	6	3745	chr6	12002985	NA	NA	0.038	16	NA	0.003	NA	9	Worcester Pearmain	-0.001	0.046	0.00004
Scarf	SNP_FB_0234424	6	3764	chr14	16827314	NA	NA	0.043	7	Jonathan	0.003	0.049	2	Ralls Janet	-0.002	0.076	0.00005
Scarf	SNP_FB_1008752	6	3765	NA	NA	Chr06	24547994	0.038	7	Jonathan	0.002	0.049	2	Ralls Janet	-0.001	0.076	0.00004
Scarf	SNP_FB_1008755	6	3766	NA	NA	Chr06	24547403	0.040	7	Jonathan	0.002	0.049	5	Golden Delicious	-0.001	0.189*	0.00004
Scarf	SNP_FB_1008766	6	3768	NA	NA	Chr06	24544801	0.044	7	Jonathan	0.003	0.049	9	Worcester Pearmain	-0.002	0.062	0.00005
Scarf	SNP_FB_1116730	6	3769	NA	NA	Chr06	24459972	0.050	7	Jonathan	0.003	0.049	2	Ralls Janet	-0.003	0.076	0.00007
Scarf	SNP_FB_0093665	8	5123	chr11	31395060	Chr08	23607516	0.040	1	Ralls Janet	0.007	0.005	7	Jonathan	-0.002	0.035	0.00003
Scarf	SNP_FB_1096133	8	5128	chr5	32324646	Chr08	23728282	0.044	1	Ralls Janet	0.006	0.005	6	Golden Delicious	-0.002	0.146	0.00004
Scarf	SNP_FB_1096139	8	5130	chr5	32325277	Chr08	23727651	0.047	1	Ralls Janet	0.007	0.005	7	Jonathan	-0.002	0.035	0.00004
Scarf	SNP_FB_0759171	8	5141	chr8	28632351	Chr08	24689343	0.045	1	Ralls Janet	0.006	0.005	7	Jonathan	-0.002	0.035	0.00004
CraTop	SNP_FB_0128827	12	7717	chr12	16984580	Chr12	16821298	0.044	13	Cox's Orange Pippin	0.001	0.030	2	Ralls Janet	-0.006	0.108*	0.00005
CraTop	SNP_FB_0129369	12	7722	chr12	17310616	NA	NA	0.050	16	NA	0.001	NA	2	Ralls Janet	-0.007	0.108*	0.00007
CraTop	SNP_FB_0129370	12	7723	chr12	17310727	NA	NA	0.045	16	NA	0.001	NA	2	Ralls Janet	-0.005	0.108*	0.00006
CraTop	SNP_FB_0129389	12	7728	chr12	17314480	NA	NA	0.039	16	NA	0.001	NA	2	Ralls Janet	-0.004	0.108*	0.00005
CraTop	GDsnp00076	15	9617	NA	NA	Chr15	9754910	0.040	5	Golden Delicious	0.003	0.141	16	NA	-0.001	NA	0.00003
CraTop	RosBREEDSNP_SNP_CA_6893289_Lg15_snpDT001928_MAF30_1622091_exon1	15	9618	chr15	6893290	Chr15	9957858	0.053	5	Golden Delicious	0.004	0.141	4	Delicious	-0.002	0.176*	0.00005
CraTop	SNP_FB_0270928	15	9620	chr15	6897556	Chr15	9962124	0.045	5	Golden Delicious	0.004	0.146	4	Delicious	-0.002	0.176*	0.00004
CraTop	SNP_FB_1072052	15	9621	NA	NA	Chr15	10028303	0.043	5	Golden Delicious	0.004	0.146	16	NA	-0.002	NA	0.00004
CraTop	SNP_FB_1072053	15	9622	NA	NA	Chr15	10028951	0.077	5	Golden Delicious	0.007	0.146	15	NA	-0.003	NA	0.00013
CraTop	RosBREEDSNP_SNP_TC_1336185_Lg16_01600_MAF40_162269_exon1	16	10566	chr16	1336185	Chr16	3157479	0.082	3	Delicious	0.011	0.165*	5	Golden Delicious	-0.011	0.149	0.00062
CraTop	SNP_FB_0335534	16	10569	chr16	1338388	Chr16	3159680	0.056	3	Delicious	0.007	0.165*	5	Golden Delicious	-0.008	0.149	0.00028
CraTop	SNP_FB_0918595	16	10572	NA	NA	Chr16	3186825	0.471	3	Delicious	0.068	0.165*	5	Golden Delicious	-0.077	0.143	0.02232
CraTop	RosBREEDSNP_SNP_AG_1538074_Lg16_LAR1_MAF40_751854_exon1	16	10577	chr16	1538074	Chr16	3407284	0.277	3	Delicious	0.034	0.165*	5	Golden Delicious	-0.041	0.149	0.00704
CraTop	RosBREEDSNP_SNP_AG_1929917_Lg16_00047_MAF40_214969_exon1	16	10589	chr16	1929917	NA	NA	0.116	3	Delicious	0.014	0.165*	5	Golden Delicious	-0.017	0.141	0.00107

Supplementary Table S3. (*Continued*)

Trait	Marker name	Chromosome	Order	GD Genome v1.0		GDDH13 Genome v1.1		Posterior prob. of QTL (γ)	Founder haplotypes with the largest or smallest genetic effects								Contribution rate
									Positive (the largest effect)				Negative (the smallest effect)				
				Chromosome	Position (bp)	Chromosome	Position (bp)		Founder haplotype	Founder	genetic effect (β)	Frequency in parental population	Founder haplotype	Founder	genetic effect (β)	Frequency in parental population	
Drop	RosBREEDSNP_SNP_GA_22208785_Lg4_01026_MAF50_1688406_exon3	4	2523	chr4	22208788	Chr04	29191185	0.043	10	Worcester Pearmain	0.007	0.038	2	Ralls Janet	-0.007	0.084	0.00004
Drop	SNP_FB_1069220	15	10019	chr14	29324904	Chr15	28941019	1.000	12	Indo	0.927	0.016	2	Ralls Janet	-0.527	0.038	0.14695
Juice	SNP_FB_0904740	4	2037	NA	NA	Chr04	2658792	0.037	2	Ralls Janet	0.007	0.097	11	Indo	-0.002	0.022	0.00003
WatCore	RosBREEDSNP_SNP_CT_13641108_Lg2_00214_MAF40_MDP0000281231_exon6	2	885	chr2	13641109	Chr02	11939271	0.032	3	Delicious	0.008	0.038	4	Delicious	-0.003	0.100	0.00004
WatCore	SNP_FB_0469344	2	907	chr2	14229127	Chr02	12526403	0.036	3	Delicious	0.009	0.038	4	Delicious	-0.003	0.100	0.00004
WatCore	SNP_FB_0469786	2	915	chr2	14482763	Chr02	12761745	0.029	3	Delicious	0.007	0.038	2	Ralls Janet	-0.004	0.124**	0.00002
WatCore	SNP_FB_0471591	2	942	chr2	15478238	Chr02	13721999	0.037	3	Delicious	0.008	0.038	4	Delicious	-0.004	0.100	0.00004
WatCore	RosBREEDSNP_SNP_GT_16174412_Lg2_01309_MAF30_MDP0000197883_exon1	2	947	chr2	16174413	Chr02	13917921	0.042	3	Delicious	0.010	0.038	4	Delicious	-0.004	0.100	0.00005
WatCore	SNP_FB_0472612	2	954	chr2	16485741	Chr02	14217371	0.028	3	Delicious	0.006	0.038	4	Delicious	-0.003	0.100	0.00002
WatCore	SNP_FB_0474201	2	985	chr2	17183905	Chr02	14855267	0.051	3	Delicious	0.013	0.035	4	Delicious	-0.006	0.081	0.00008
WatCore	RosBREEDSNP_SNP_CA_23859847_Lg14_327428_MAF20_327428_exon1	14	9209	chr14	23859849	Chr14	24131385	0.047	4	Delicious	0.007	0.114	6	Golden Delicious	-0.004	0.249**	0.00008
WatCore	SNP_FB_0245797	14	9215	chr14	23862009	Chr14	24133545	0.041	4	Delicious	0.007	0.114	6	Golden Delicious	-0.004	0.249**	0.00007
WatCore	SNP_FB_0245802	14	9217	chr14	23863565	Chr14	24135106	0.031	4	Delicious	0.005	0.114	6	Golden Delicious	-0.002	0.249**	0.00004
WatCore	SNP_FB_0246451	14	9218	chr14	24795847	Chr14	24278265	0.141	4	Delicious	0.025	0.114	6	Golden Delicious	-0.013	0.249**	0.00101
WatCore	SNP_FB_1097692	14	9221	chr14	24799135	Chr06	27773751	0.033	4	Delicious	0.005	0.114	6	Golden Delicious	-0.003	0.249**	0.00005
WatCore	RosBREEDSNP_SNP_CA_24046950_Lg14_01531_MAF20_1658676_exon2	14	9227	chr14	24047881	Chr14	24374286	0.058	4	Delicious	0.010	0.114	1	Ralls Janet	-0.007	0.008	0.00016
WatCore	SNP_FB_0246016	14	9229	chr14	24050291	Chr14	24376695	0.029	4	Delicious	0.005	0.114	1	Ralls Janet	-0.003	0.008	0.00004
WatCore	SNP_FB_0246017	14	9230	chr14	24050502	Chr14	24376906	0.045	4	Delicious	0.008	0.114	6	Golden Delicious	-0.004	0.249**	0.00009
WatCore	SNP_FB_0246018	14	9231	chr14	24050509	Chr14	24376913	0.029	4	Delicious	0.005	0.114	1	Ralls Janet	-0.004	0.008	0.00003
WatCore	RosBREEDSNP_SNP_AC_24054772_Lg14_01531_MAF30_255324_exon1	14	9236	chr14	24055703	Chr14	24382115	0.030	4	Delicious	0.005	0.114	1	Ralls Janet	-0.002	0.008	0.00004
WatCore	SNP_FB_0247524	14	9245	chr14	26124982	Chr14	24740012	0.027	4	Delicious	0.005	0.114	9	Worcester Pearmain	-0.002	0.065	0.00003
WatCore	SNP_FB_0247841	14	9270	chr14	26601783	Chr14	25388076	0.031	4	Delicious	0.006	0.111	6	Golden Delicious	-0.002	0.262**	0.00004
WatCore	SNP_FB_0247873	14	9275	chr14	26608317	Chr14	25394670	0.055	4	Delicious	0.009	0.111	15	NA	-0.004	NA	0.00014
Acidity	RosBREEDSNP_SNP_GA_16535826_Lg8_02022_MAF20_1637535_exon1	8	4859	chr8	16535827	Chr08	10068536	1.000	7	Jonathan	0.499	0.038	3	Delicious	-0.385	0.024	0.13892
Acidity	SNP_FB_0334898	16	10543	chr16	950565	NA	NA	0.031	1	Ralls Janet	0.007	0.003**	2	Ralls Janet	-0.002	0.054	0.00001
Acidity	SNP_FB_0918592	16	10571	NA	NA	Chr16	3186476	1.000	1	Ralls Janet	0.882	0.003**	11	Indo	-0.474	0.011	0.15666
Weight	SNP_FB_0640513	5	3277	chr5	33261787	Chr05	16812370	0.040	5	Golden Delicious	0.494	0.076	13	Cox's Orange Pippin	-0.321	0.059	0.00004
Weight	SNP_FB_0640524	5	3279	chr5	33264272	Chr05	16809885	0.030	2	Ralls Janet	0.438	0.114*	12	Indo	-0.264	0.030	0.00002
Weight	SNP_FB_0152382	12	8068	chr12	34290036	Chr12	30895746	0.045	4	Delicious	0.417	0.105	2	Ralls Janet	-0.644	0.105	0.00003
Weight	SNP_FB_0160788	13	8243	chr13	2450777	Chr13	2827672	0.039	1	Ralls Janet	0.334	0.011	3	Delicious	-0.296	0.019**	0.00002
Weight	SNP_FB_1063136	13	8246	chr16	1382603	Chr16	3205361	0.039	1	Ralls Janet	0.423	0.011	3	Delicious	-0.328	0.019**	0.00002
Weight	SNP_FB_1063138	13	8248	NA	NA	Chr13	3094815	0.029	1	Ralls Janet	0.274	0.011	6	Golden Delicious	-0.259	0.135	0.00001
Weight	SNP_FB_1063148	13	8249	NA	NA	Chr13	3097517	0.029	4	Delicious	0.182	0.159*	3	Delicious	-0.226	0.019**	0.00001
Weight	GDsnp00195	13	8250	NA	NA	Chr13	3222832	0.035	8	Jonathan	0.312	0.208**	3	Delicious	-0.339	0.019**	0.00002
Weight	SNP_FB_0161441	13	8253	chr13	2831697	Chr13	3226727	0.035	4	Delicious	0.280	0.159*	6	Golden Delicious	-0.305	0.135	0.00002
Weight	SNP_FB_0161448	13	8257	chr13	2833298	Chr13	3228329	0.030	8	Jonathan	0.229	0.208**	3	Delicious	-0.268	0.022**	0.00001
Weight	RosBREEDSNP_SNP_GA_1216243_Lg16_01588_MAF20_468133_exon1	16	10553	chr16	1216243	Chr16	3025066	0.032	2	Ralls Janet	0.272	0.054	8	Jonathan	-0.434	0.146*	0.00001
Weight	RosBREEDSNP_SNP_AG_1538074_Lg16_LAR1_MAF40_751854_exon1	16	10577	chr16	1538074	Chr16	3407284	0.043	2	Ralls Janet	0.314	0.054	8	Jonathan	-0.504	0.151*	0.00003
Weight	RosBREEDSNP_SNP_CT_1540624_Lg16_LAR1_MAF40_1618769_exon2	16	10578	chr16	1540624	Chr16	3409834	0.031	15	NA	0.193	NA	8	Jonathan	-0.341	0.151*	0.00001
Weight	RosBREEDSNP_SNP_TC_1571002_Lg16_01734_MAF40_216397_exon1	16	10579	chr16	1571002	Chr16	3439690	0.056	16	NA	0.636	NA	8	Jonathan	-0.709	0.151*	0.00005
Weight	RosBREEDSNP_SNP_GT_1578941_Lg16_01734_MAF20_90585_exon1	16	10580	chr16	1578941	Chr16	3447621	0.031	1	Ralls Janet	0.213	0.003**	8	Jonathan	-0.361	0.151*	0.00001
Weight	SNP_FB_0336036	16	10581	NA	NA	Chr16	3479443	0.054	15	NA	0.555	NA	8	Jonathan	-0.729	0.151*	0.00004
Weight	SNP_FB_0336046	16	10582	NA	NA	Chr16	3482544	0.040	15	NA	0.347	NA	8	Jonathan	-0.525	0.151*	0.00002
Weight	SNP_FB_0336063	16	10583	chr16	1617578	Chr16	3486139	0.063	2	Ralls Janet	0.450	0.054	8	Jonathan	-0.861	0.151*	0.00006
Weight	RosBREEDSNP_SNP_TC_1669091_Lg16_01734_MAF50_193795_exon1	16	10586	chr16	1669091	Chr16	3556998	0.037	15	NA	0.356	NA	8	Jonathan	-0.389	0.151*	0.00002
Weight	SNP_FB_1074699	16	10588	NA	NA	Chr16	3774681	0.032	16	NA	0.294	NA	8	Jonathan	-0.321	0.149*	0.00001
Brix	SNP_FB_1075309	16	10728	NA	NA	Chr16	7832509	0.037	12	Indo	0.030	0.008	1	Ralls Janet	-0.043	0.003**	0.00007
Brix	SNP_FB_0921308	16	10731	NA	NA	Chr16	7913908	0.049	12	Indo	0.037	0.008	1	Ralls Janet	-0.043	0.003**	0.00013
Brix	SNP_FB_0344904	16	10735	chr16	6005835	Chr16	8230735	0.069	12	Indo	0.054	0.008	1	Ralls Janet	-0.047	0.003**	0.00026
Brix	SNP_FB_0344908	16	10737	chr16	6006173	Chr16	8231073	0.069	12	Indo	0.054	0.008	1	Ralls Janet	-0.052	0.003**	0.00025
Brix	RosBREEDSNP_SNP_GA_6007512_Lg16_01003_MAF50_MDP0000188823_exon2	16	10739	chr16	6007512	Chr16	8232408	0.149	12	Indo	0.120	0.008	1	Ralls Janet	-0.140	0.003**	0.00127
Brix	SNP_FB_0344930	16	10740	chr16	6010460	Chr16	8235344	0.061	12	Indo	0.049	0.008	1	Ralls Janet	-0.060	0.003**	0.00021
Brix	SNP_FB_0345412	16	10744	chr16	6257493	Chr16	8475717	0.080	12	Indo	0.061	0.008	1	Ralls Janet	-0.069	0.003**	0.00034
Brix	SNP_FB_0345423	16	10748	chr16	6260819	Chr16	8479050	0.055	12	Indo	0.039	0.008	1	Ralls Janet	-0.031	0.003**	0.00014
Brix	SNP_FB_1075559	16	10755	NA	NA	Chr16	8848360	0.031	12	Indo	0.025	0.008	1	Ralls Janet	-0.025	0.003**	0.00005
Brix	RosBREEDSNP_SNP_TC_6556419_Lg16_01866_MAF20_MDP0000291589_exon2	16	10760	chr16	6556419	NA	NA	0.063	12	Indo	0.052	0.008	1	Ralls Janet	-0.055	0.003**	0.00022
Brix	SNP_FB_0921783	16	10762	chr16	6559479	Chr16	8854667	0.056	12	Indo	0.046	0.008	1	Ralls Janet	-0.043	0.003**	0.00017
Brix	RosBREEDSNP_SNP_TC_6842969_Lg16_02194_MAF50_27319_exon1	16	10770	chr16	6842969	Chr16	9125644	0.039	12	Indo	0.024	0.008	1	Ralls Janet	-0.021	0.003**	0.00006
Brix	RosBREEDSNP_SNP_GA_6845933_Lg16_02194_MAF50_390497_exon1	16	10771	chr16	6845933	Chr16	9128609	0.034	12	Indo	0.019	0.008	1	Ralls Janet	-0.018	0.003**	0.00004

Supplementary Table S3. (*Continued*)

Trait	Marker name	Chromosome	Order	GD Genome v1.0		GDDH13 Genome v1.1		Posterior prob. of QTL (γ)	Founder haplotypes with the largest or smallest genetic effects								Contribution rate
									Positive (the largest effect)				Negative (the smallest effect)				
				Chromosome	Position (bp)	Chromosome	Position (bp)		Founder haplotype	Founder	genetic effect (β)	Frequency in parental population	Founder haplotype	Founder	genetic effect (β)	Frequency in parental population	
MalAcid	SNP_FB_0741253	8	4850	chr8	14331923	Chr08	9726043	0.070	9	Worcester Pearmain	0.005	0.014	3	Delicious	-0.007	0.024	0.00035
MalAcid	RosBREEDSNP_SNP_CT_16509379_Lg8_324685_MAF40_324685_exon1	8	4858	chr8	16509380	Chr15	6433343	0.050	7	Jonathan	0.004	0.038	3	Delicious	-0.004	0.024	0.00023
MalAcid	SNP_FB_0745806	8	4864	chr8	16590075	Chr08	10134680	0.063	7	Jonathan	0.006	0.038	3	Delicious	-0.007	0.024	0.00046
MalAcid	SNP_FB_0746385	8	4870	chr8	16942507	Chr08	10613040	0.075	7	Jonathan	0.007	0.038	3	Delicious	-0.007	0.024	0.00063
MalAcid	SNP_FB_0746401	8	4871	chr8	16944888	Chr08	10615421	0.092	7	Jonathan	0.008	0.038	3	Delicious	-0.010	0.024	0.00108
MalAcid	SNP_FB_0746410	8	4872	chr8	16947009	Chr08	10617538	0.096	7	Jonathan	0.009	0.038	3	Delicious	-0.009	0.024	0.00107
MalAcid	RosBREEDSNP_SNP_CT_17343145_Lg8_01370_MAF20_MDP0000769686_exon3	8	4873	chr8	17343146	Chr08	11006715	0.044	7	Jonathan	0.004	0.038	3	Delicious	-0.004	0.024	0.00020
MalAcid	RosBREEDSNP_SNP_CT_17361806_Lg8_01370_MAF10_MDP0000220614_exon3	8	4874	chr8	17361807	Chr08	11027624	0.104	7	Jonathan	0.009	0.038	3	Delicious	-0.010	0.024	0.00104
MalAcid	SNP_FB_0747616	8	4877	chr8	17388150	Chr08	11053814	0.055	7	Jonathan	0.004	0.038	3	Delicious	-0.005	0.024	0.00023
MalAcid	RosBREEDSNP_SNP_TC_1336185_Lg16_01600_MAF40_162269_exon1	16	10566	chr16	1336185	Chr16	3157479	0.061	1	Ralls Janet	0.006	0.003**	11	Indo	-0.005	0.011	0.00027
MalAcid	SNP_FB_0335525	16	10568	chr16	1336595	Chr16	3157889	0.092	1	Ralls Janet	0.008	0.003**	11	Indo	-0.008	0.011	0.00070
MalAcid	SNP_FB_0918592	16	10571	NA	NA	Chr16	3186476	0.179	1	Ralls Janet	0.025	0.003**	11	Indo	-0.023	0.011	0.00538
MalAcid	SNP_FB_0918595	16	10572	NA	NA	Chr16	3186825	0.121	1	Ralls Janet	0.024	0.003**	11	Indo	-0.018	0.011	0.00270
MalAcid	RosBREEDSNP_SNP_AC_1452699_Lg16_MDP0000303483_MAF50_MDP0000303483_exon2	16	10573	chr16	1452699	Chr16	3304530	0.091	1	Ralls Janet	0.011	0.003**	11	Indo	-0.012	0.011	0.00134
MalAcid	SNP_FB_1074682	16	10575	NA	NA	Chr16	3362846	0.054	1	Ralls Janet	0.008	0.003**	11	Indo	-0.007	0.011	0.00048
MalAcid	RosBREEDSNP_SNP_TC_1496083_Lg16_LAR1_MAF30_MDP0000279135_exon2	16	10576	chr16	1496083	Chr16	3349986	0.061	1	Ralls Janet	0.008	0.003**	11	Indo	-0.008	0.011	0.00064
MalAcid	RosBREEDSNP_SNP_AG_1538074_Lg16_LAR1_MAF40_751854_exon1	16	10577	chr16	1538074	Chr16	3407284	0.137	1	Ralls Janet	0.018	0.003**	11	Indo	-0.014	0.011	0.00182
MalAcid	RosBREEDSNP_SNP_TC_1571002_Lg16_01734_MAF40_216397_exon1	16	10579	chr16	1571002	Chr16	3439690	0.218	1	Ralls Janet	0.031	0.003**	11	Indo	-0.027	0.011	0.00582
MalAcid	RosBREEDSNP_SNP_GT_1578941_Lg16_01734_MAF20_90585_exon1	16	10580	chr16	1578941	Chr16	3447621	0.058	1	Ralls Janet	0.008	0.003**	11	Indo	-0.008	0.011	0.00049
MalAcid	GDsnp01734	16	10585	chr16	1620546	Chr16	3489107	0.044	1	Ralls Janet	0.006	0.003**	11	Indo	-0.005	0.011	0.00022
Firm	SNP_FB_0546485	3	1873	chr3	32029629	Chr03	31326611	0.057	7	Jonathan	0.034	0.151*	9	Worcester Pearmain	-0.025	0.038	0.00006
Firm	SNP_FB_0546508	3	1877	chr3	32034719	Chr03	31331715	0.056	7	Jonathan	0.035	0.154*	9	Worcester Pearmain	-0.029	0.038	0.00007
Firm	SNP_FB_0546521	3	1878	chr3	32036453	Chr03	31333294	0.058	7	Jonathan	0.039	0.154*	9	Worcester Pearmain	-0.026	0.038	0.00008
Firm	SNP_FB_0546805	3	1881	chr3	32312727	Chr03	31597278	0.051	7	Jonathan	0.033	0.151*	9	Worcester Pearmain	-0.029	0.038	0.00007
Firm	SNP_FB_0546827	3	1886	chr3	32317504	Chr03	31602090	0.051	7	Jonathan	0.028	0.151*	9	Worcester Pearmain	-0.026	0.038	0.00005
Firm	SNP_FB_0546828	3	1887	chr3	32317699	Chr03	31602285	0.052	7	Jonathan	0.034	0.151*	9	Worcester Pearmain	-0.024	0.038	0.00005
Firm	SNP_FB_0058572	11	6770	chr11	4627473	NA	NA	0.239	11	Indo	0.251	0.022	5	Golden Delicious	-0.192	0.030**	0.00381
Firm	SNP_FB_0058955	11	6771	chr11	4923989	Chr11	4166925	0.125	8	Jonathan	0.123	0.068	5	Golden Delicious	-0.111	0.030**	0.00100
Firm	SNP_FB_0059435	11	6786	chr11	5163288	Chr11	4436899	0.098	11	Indo	0.093	0.022	5	Golden Delicious	-0.095	0.030**	0.00062
Firm	RosBREEDSNP_SNP_AC_5173582_Lg11_00418_MAF20_719249_exon1	11	6788	chr11	5173582	Chr11	4484758	0.201	11	Indo	0.201	0.022	5	Golden Delicious	-0.192	0.030**	0.00290
Firm	RosBREEDSNP_SNP_CT_5482687_Lg11_01713_MAF40_MDP0000265419_exon1	11	6796	chr11	5482687	Chr11	4780438	0.067	8	Jonathan	0.060	0.062	5	Golden Delicious	-0.060	0.030**	0.00027
Firm	SNP_FB_0061199	11	6801	chr11	6190208	Chr11	5436992	0.117	11	Indo	0.118	0.022	5	Golden Delicious	-0.103	0.030**	0.00097
Firm	SNP_FB_0166956	13	8356	chr13	6365657	Chr13	6765583	0.333	16	NA	0.409	NA	15	NA	-0.338	NA	0.00345
Firm	SNP_FB_0168230	13	8380	chr13	7297873	Chr13	7732961	0.158	15	NA	0.167	NA	16	NA	-0.131	NA	0.00062
Firm	SNP_FB_0168770	13	8393	chr13	7587457	Chr13	8002993	0.076	15	NA	0.086	NA	16	NA	-0.058	NA	0.00013
Firm	SNP_FB_0895019	14	9321	chr14	29195272	Chr14	27609717	0.051	3	Delicious	0.035	0.192**	1	Ralls Janet	-0.039	0.011	0.00005
Firm	RosBREEDSNP_SNP_AG_1516126_Lg15_00717_MAF40_530377_exon1	15	9523	chr15	1516127	Chr15	4724375	0.146	2	Ralls Janet	0.076	0.005	16	NA	-0.117	NA	0.00047
DegMeal	SNP_FB_1052855	10	6220	NA	NA	Chr10	14877221	0.043	3	Delicious	0.005	0.019**	15	NA	-0.005	NA	0.00014
DegMeal	SNP_FB_0024056	10	6334	chr10	17882677	Chr10	24446681	0.079	3	Delicious	0.009	0.016**	5	Golden Delicious	-0.006	0.186	0.00033
DegMeal	SNP_FB_0026018	10	6365	chr10	18871174	Chr10	25567242	0.042	3	Delicious	0.005	0.016**	1	Ralls Janet	-0.003	0.089	0.00009
DegMeal	SNP_FB_0028767	10	6396	chr10	20053833	Chr10	26825052	0.103	16	NA	0.011	NA	15	NA	-0.011	NA	0.00081
DegMeal	SNP_FB_0028781	10	6399	chr10	20057099	Chr10	26828318	0.069	16	NA	0.008	NA	1	Ralls Janet	-0.007	0.086	0.00037
MaxMeal	SNP_FB_0546521	3	1878	chr3	32036453	Chr03	31333294	0.039	8	Jonathan	0.002	0.027	1	Ralls Janet	-0.005	0.014	0.00004
MaxMeal	SNP_FB_0546805	3	1881	chr3	32312727	Chr03	31597278	0.048	8	Jonathan	0.002	0.027	1	Ralls Janet	-0.005	0.014	0.00005
MaxMeal	SNP_FB_0974555	3	1889	NA	NA	Chr03	32252840	0.030	3	Delicious	0.001	0.122	1	Ralls Janet	-0.002	0.011	0.00001
MaxMeal	RosBREEDSNP_SNP_TC_32157764_Lg10_257441__257441_exon2	10	6580	chr10	32157765	Chr10	37603889	0.031	9	Worcester Pearmain	0.004	0.054	6	Golden Delicious	-0.002	0.041**	0.00006
MaxMeal	RosBREEDSNP_SNP_GT_32395620_Lg10_RosCOS2362_MAF30_1644148_exon2	10	6584	chr10	32395621	Chr10	37832244	0.042	9	Worcester Pearmain	0.005	0.054	6	Golden Delicious	-0.002	0.041**	0.00010
MaxMeal	RosBREEDSNP_SNP_CT_32402084_Lg10_RosCOS2362_MAF40_MDP0000383515_exon5	10	6585	chr10	32402085	Chr10	37838711	0.091	9	Worcester Pearmain	0.013	0.054	6	Golden Delicious	-0.005	0.041**	0.00055
MaxMeal	SNP_FB_0045158	10	6591	chr10	32755261	Chr10	38240726	0.036	9	Worcester Pearmain	0.004	0.054	5	Golden Delicious	-0.002	0.214**	0.00006
MaxMeal	SNP_FB_0046460	10	6596	chr10	33483574	Chr10	38974048	0.034	9	Worcester Pearmain	0.003	0.062	5	Golden Delicious	-0.002	0.211**	0.00005
MaxMeal	RosBREEDSNP_SNP_TC_33513449_Lg10_01683_MAF30_1630378_exon3	10	6599	chr10	33513450	Chr10	39003912	0.032	9	Worcester Pearmain	0.004	0.062	1	Ralls Janet	-0.002	0.070	0.00004

Supplementary Table S3. (*Continued*)

Trait	Marker name	Chromosome	Order	GD Genome v1.0 Chromosome	GD Genome v1.0 Position (bp)	GDDH13 Genome v1.1 Chromosome	GDDH13 Genome v1.1 Position (bp)	Posterior prob. of QTL (γ)	Positive (the largest effect) Founder haplotype	Positive Founder	Positive genetic effect (β)	Positive Frequency in parental population	Negative (the smallest effect) Founder haplotype	Negative Founder	Negative genetic effect (β)	Negative Frequency in parental population	Contribution rate
Suc	SNP_FB_0402047	17	11707	chr17	21335611	Chr17	28631021	0.060	16	NA	1.114	NA	10	Worcester Pearmain	-1.877	0.035	0.00002
Suc	SNP_FB_0402605	17	11716	chr17	21757680	Chr17	29069166	0.050	7	Jonathan	1.526	0.116	10	Worcester Pearmain	-2.642	0.032	0.00003
Suc	SNP_FB_0402631	17	11722	chr17	21762555	Chr17	29074039	0.054	7	Jonathan	1.733	0.116	10	Worcester Pearmain	-2.599	0.032	0.00003
Suc	SNP_FB_1112873	17	11734	NA	NA	Chr17	29333596	0.055	7	Jonathan	1.398	0.116	10	Worcester Pearmain	-3.333	0.030	0.00003
Suc	SNP_FB_0937353	17	11737	chr17	23264319	Chr17	30074499	0.055	7	Jonathan	1.414	0.119	10	Worcester Pearmain	-3.287	0.030	0.00003
Suc	SNP_FB_0937354	17	11740	NA	NA	Chr17	30074513	0.053	7	Jonathan	1.382	0.119	10	Worcester Pearmain	-2.663	0.030	0.00002
Suc	RosBREEDSNP_SNP_AC_24577383_Lg17_02678_MAF10_179861_exon2	17	11746	chr17	24599088	Chr02	17358537	0.049	7	Jonathan	1.183	0.122	10	Worcester Pearmain	-2.213	0.030	0.00002
Suc	SNP_FB_0406116	17	11751	chr17	25075514	Chr17	32330312	0.049	7	Jonathan	1.186	0.122	10	Worcester Pearmain	-1.891	0.030	0.00002
Suc	SNP_FB_0406851	17	11754	chr17	25363505	Chr17	32629327	0.051	2	Ralls Janet	1.171	0.019	10	Worcester Pearmain	-2.081	0.030	0.00002
Suc	SNP_FB_0406852	17	11755	chr17	25363811	Chr17	32629633	0.054	7	Jonathan	1.076	0.122	10	Worcester Pearmain	-2.547	0.030	0.00003
Suc	SNP_FB_0406865	17	11758	chr17	25365200	Chr17	32631021	0.060	2	Ralls Janet	1.184	0.019	10	Worcester Pearmain	-2.833	0.030	0.00003
Suc	SNP_FB_0406871	17	11759	chr17	25366876	Chr17	32632705	0.056	16	NA	1.592	NA	10	Worcester Pearmain	-2.570	0.030	0.00002
Suc	RosBREEDSNP_SNP_GT_25452621_Lg17_01467_MAF50_236982_exon1	17	11762	chr17	25452621	Chr17	32713992	0.049	13	Cox's Orange Pippin	0.745	0.089**	10	Worcester Pearmain	-2.111	0.030	0.00002
Suc	RosBREEDSNP_SNP_AG_25527294_Lg17_01467_MAF10_1646394_exon4	17	11763	chr17	25527294	Chr17	32777061	0.087	16	NA	2.042	NA	10	Worcester Pearmain	-4.401	0.030	0.00008
Suc	RosBREEDSNP_SNP_CA_25607759_Lg17_00809_MAF50_221015_exon1	17	11765	chr17	25607759	Chr17	32900869	0.072	7	Jonathan	1.289	0.114	10	Worcester Pearmain	-3.921	0.030	0.00005
Suc	SNP_FB_0408375	17	11769	chr17	26062664	Chr17	33419814	0.053	2	Ralls Janet	1.236	0.019	10	Worcester Pearmain	-2.924	0.030	0.00002
Suc	SNP_FB_0408392	17	11771	chr17	26066069	Chr17	33423246	0.055	7	Jonathan	0.978	0.116	10	Worcester Pearmain	-2.532	0.030	0.00002
Suc	SNP_FB_1112978	17	11777	NA	NA	Chr17	33782069	0.058	7	Jonathan	0.688	0.116	10	Worcester Pearmain	-2.287	0.030	0.00002
Suc	SNP_FB_1112979	17	11778	NA	NA	Chr17	33782300	0.057	2	Ralls Janet	1.539	0.019	10	Worcester Pearmain	-2.761	0.030	0.00003
Suc	SNP_FB_1112996	17	11779	NA	NA	Chr17	33788733	0.058	7	Jonathan	0.973	0.116	10	Worcester Pearmain	-2.146	0.030	0.00002
Suc	SNP_FB_0408804	17	11782	chr17	26483899	Chr17	33892609	0.056	2	Ralls Janet	2.102	0.019	10	Worcester Pearmain	-2.537	0.030	0.00003
Glu	SNP_FB_0980766	4	2255	NA	NA	Chr04	17923983	0.046	2	Ralls Janet	0.821	0.119**	5	Golden Delicious	-0.176	0.168	0.00006
Glu	SNP_FB_0864902	12	7914	NA	NA	Chr12	26743264	0.054	16	NA	0.930	NA	15	NA	-0.750	NA	0.00009
Glu	RosBREEDSNP_SNP_GA_21188396_Lg17_00423_MAF50_MDP0000538106_exon1	17	11697	chr17	21188396	Chr17	28496419	0.047	2	Ralls Janet	0.419	0.019	10	Worcester Pearmain	-0.436	0.035	0.00004
Glu	SNP_FB_0402050	17	11708	chr17	21336292	Chr17	28631702	0.034	2	Ralls Janet	0.303	0.019	10	Worcester Pearmain	-0.334	0.035	0.00002
Glu	RosBREEDSNP_SNP_AG_21759969_Lg17_RosCOS2078_MAF30_549398_exon1	17	11719	chr17	21759969	Chr17	29071454	0.032	7	Jonathan	0.214	0.116	10	Worcester Pearmain	-0.461	0.032	0.00002
Glu	SNP_FB_0402626	17	11721	chr17	21760780	Chr17	29072265	0.032	7	Jonathan	0.256	0.116	10	Worcester Pearmain	-0.527	0.032	0.00002
Glu	RosBREEDSNP_SNP_TC_21762855_Lg17_RosCOS2078_MAF20_MDP0000292200_exon11	17	11723	chr17	21762855	Chr17	29074339	0.034	16	NA	0.251	NA	10	Worcester Pearmain	-0.544	0.032	0.00003
Glu	RosBREEDSNP_SNP_TC_21775385_Lg17_RosCOS2078_MAF30_266423_exon1	17	11724	chr17	21775385	Chr17	29094710	0.035	15	NA	0.260	NA	10	Worcester Pearmain	-0.460	0.032	0.00003
Glu	SNP_FB_0840402	17	11727	chr17	21830528	Chr17	29206609	0.040	9	Worcester Pearmain	0.364	0.008	10	Worcester Pearmain	-0.625	0.032	0.00003
Glu	GDsnp02678	17	11738	chr17	24545250	Chr17	31724902	0.033	7	Jonathan	0.248	0.119	10	Worcester Pearmain	-0.557	0.030	0.00003
Glu	SNP_FB_0937354	17	11740	NA	NA	Chr17	30074513	0.034	7	Jonathan	0.312	0.119	10	Worcester Pearmain	-0.436	0.030	0.00003
Glu	SNP_FB_0405253	17	11741	chr17	24575077	Chr17	31758477	0.032	9	Worcester Pearmain	0.253	0.008	10	Worcester Pearmain	-0.446	0.030	0.00002
Glu	SNP_FB_0405232	17	11744	chr17	24596857	Chr17	31752415	0.038	2	Ralls Janet	0.280	0.019	10	Worcester Pearmain	-0.596	0.030	0.00004
Glu	RosBREEDSNP_SNP_AC_24577383_Lg17_02678_MAF10_179861_exon2	17	11746	chr17	24599088	Chr02	17358537	0.035	9	Worcester Pearmain	0.165	0.008	10	Worcester Pearmain	-0.507	0.030	0.00002
Glu	SNP_FB_0406865	17	11758	chr17	25365200	Chr17	32631021	0.052	2	Ralls Janet	0.569	0.019	10	Worcester Pearmain	-0.704	0.030	0.00006
Glu	RosBREEDSNP_SNP_GT_25452621_Lg17_01467_MAF50_236982_exon1	17	11762	chr17	25452621	Chr17	32713992	0.033	2	Ralls Janet	0.295	0.019	10	Worcester Pearmain	-0.465	0.030	0.00002
Glu	SNP_FB_0408387	17	11770	chr17	26064671	Chr17	33421848	0.034	2	Ralls Janet	0.400	0.019	10	Worcester Pearmain	-0.489	0.030	0.00003
Glu	SNP_FB_0408394	17	11772	chr17	26066733	Chr17	33423910	0.041	2	Ralls Janet	0.626	0.019	10	Worcester Pearmain	-0.506	0.030	0.00003
Glu	SNP_FB_0408413	17	11774	chr17	26070324	Chr17	33427495	0.039	9	Worcester Pearmain	0.197	0.008	10	Worcester Pearmain	-0.581	0.030	0.00003
Glu	SNP_FB_1112996	17	11779	NA	NA	Chr17	33788733	0.040	7	Jonathan	0.182	0.116	10	Worcester Pearmain	-0.539	0.030	0.00004

Founder haplotypes with the largest or smallest genetic effects

Supplementary Table S3. (*Continued*)

Trait	Marker name	Chromosome	Order	GD Genome v1.0		GDDH13 Genome v1.1		Posterior prob. of QTL (γ)	Founder haplotypes with the largest or smallest genetic effects								Contribution rate
									Positive (the largest effect)				Negative (the smallest effect)				
				Chromosome	Position (bp)	Chromosome	Position (bp)		Founder haplotype	Founder	genetic effect (β)	Frequency in parental population	Founder haplotype	Founder	genetic effect (β)	Frequency in parental population	
Fru	SNP_FB_0402050	17	11708	chr17	21336292	Chr17	28631702	0.058	9	Worcester Pearmain	1.320	0.008	10	Worcester Pearmain	-2.793	0.035	0.00003
Fru	SNP_FB_0402591	17	11713	chr17	21755678	Chr17	29067164	0.054	7	Jonathan	1.844	0.116	10	Worcester Pearmain	-3.398	0.032	0.00004
Fru	SNP_FB_0402595	17	11714	chr17	21756411	Chr17	29067897	0.054	7	Jonathan	1.470	0.116	10	Worcester Pearmain	-3.383	0.032	0.00003
Fru	SNP_FB_0402623	17	11720	chr17	21760490	Chr17	29071975	0.063	7	Jonathan	2.114	0.116	10	Worcester Pearmain	-3.766	0.032	0.00005
Fru	RosBREEDSNP_SNP_TC_21762855_Lg17_RosCOS2078_MAF20_MDP0000292200_exon11	17	11723	chr17	21762855	Chr17	29074339	0.055	7	Jonathan	1.954	0.116	10	Worcester Pearmain	-2.561	0.032	0.00004
Fru	RosBREEDSNP_SNP_TC_21775385_Lg17_RosCOS2078_MAF30_266423_exon1	17	11724	chr17	21775385	Chr17	29094710	0.060	7	Jonathan	1.939	0.116	10	Worcester Pearmain	-3.557	0.032	0.00005
Fru	SNP_FB_1112855	17	11728	chr17	21948936	Chr17	29327604	0.054	7	Jonathan	1.811	0.116	10	Worcester Pearmain	-3.251	0.032	0.00004
Fru	SNP_FB_1112873	17	11734	NA	NA	Chr17	29333596	0.054	7	Jonathan	1.822	0.116	10	Worcester Pearmain	-3.363	0.030	0.00004
Fru	SNP_FB_0405230	17	11743	chr17	24596678	Chr17	31752236	0.062	7	Jonathan	1.694	0.122	10	Worcester Pearmain	-3.974	0.030	0.00006
Fru	SNP_FB_0405232	17	11744	chr17	24596857	Chr17	31752415	0.056	7	Jonathan	1.556	0.122	10	Worcester Pearmain	-3.409	0.030	0.00004
Fru	RosBREEDSNP_SNP_GA_25284422_Lg17_MDP0000194307_MAF10_MDP0000194307_exon13	17	11753	chr17	25284422	Chr17	32549666	0.076	9	Worcester Pearmain	1.918	0.008	10	Worcester Pearmain	-4.203	0.030	0.00009
Fru	SNP_FB_0406862	17	11757	chr17	25364864	Chr17	32630685	0.051	9	Worcester Pearmain	1.077	0.008	10	Worcester Pearmain	-2.712	0.030	0.00003
Fru	SNP_FB_0406871	17	11759	chr17	25366876	Chr17	32632705	0.061	7	Jonathan	1.576	0.122	10	Worcester Pearmain	-3.672	0.030	0.00005
Fru	SNP_FB_0406872	17	11760	chr17	25372062	Chr17	32633167	0.063	7	Jonathan	1.338	0.122	10	Worcester Pearmain	-3.588	0.030	0.00005
Fru	RosBREEDSNP_SNP_GT_25452621_Lg17_01467_MAF50_236982_exon1	17	11762	chr17	25452621	Chr17	32713992	0.052	9	Worcester Pearmain	1.339	0.008	10	Worcester Pearmain	-3.099	0.030	0.00003
Fru	GDsnp00809	17	11766	chr17	25640634	Chr17	32962200	0.051	9	Worcester Pearmain	1.125	0.008	10	Worcester Pearmain	-3.001	0.030	0.00003
Fru	SNP_FB_0938201	17	11768	NA	NA	Chr17	34701384	0.051	7	Jonathan	1.257	0.116	10	Worcester Pearmain	-2.154	0.030	0.00002
Fru	SNP_FB_0408392	17	11771	chr17	26066069	Chr17	33423246	0.076	9	Worcester Pearmain	1.918	0.008	10	Worcester Pearmain	-4.650	0.030	0.00009
Fru	SNP_FB_0408802	17	11781	chr17	26483679	Chr17	33892389	0.054	9	Worcester Pearmain	1.428	0.008	10	Worcester Pearmain	-2.953	0.030	0.00004
Fru	SNP_FB_0408810	17	11785	chr17	26485697	Chr17	33894407	0.066	2	Ralls Janet	2.073	0.019	10	Worcester Pearmain	-5.035	0.030	0.00009
Sor	SNP_FB_0918592	16	10571	NA	NA	Chr16	3186476	0.039	10	Worcester Pearmain	0.064	0.019	8	Jonathan	-0.030	0.151*	0.00007
Sor	SNP_FB_1074682	16	10575	NA	NA	Chr16	3362846	0.043	10	Worcester Pearmain	0.079	0.019	6	Golden Delicious	-0.028	0.078	0.00009
Sor	RosBREEDSNP_SNP_CT_1540624_Lg16_LAR1_MAF40_1618769_exon2	16	10578	chr16	1540624	Chr16	3409834	0.044	1	Ralls Janet	0.093	0.003**	8	Jonathan	-0.040	0.151*	0.00011
Sor	RosBREEDSNP_SNP_TC_1571002_Lg16_01734_MAF40_216397_exon1	16	10579	chr16	1571002	Chr16	3439690	0.045	10	Worcester Pearmain	0.081	0.019	8	Jonathan	-0.046	0.151*	0.00012
Sor	RosBREEDSNP_SNP_GT_1578941_Lg16_01734_MAF20_90585_exon1	16	10580	chr16	1578941	Chr16	3447621	0.041	10	Worcester Pearmain	0.075	0.019	8	Jonathan	-0.035	0.151*	0.00008
Sor	RosBREEDSNP_SNP_AG_1617869_Lg16_01734_MAF20_248174_exon1	16	10584	chr16	1617869	Chr16	3486430	0.052	10	Worcester Pearmain	0.102	0.019	15	NA	-0.050	NA	0.00017
Sor	GDsnp01734	16	10585	chr16	1620546	Chr16	3489107	0.072	10	Worcester Pearmain	0.136	0.019	8	Jonathan	-0.062	0.151*	0.00030
Sor	SNP_FB_0337026	16	10593	chr16	1968664	Chr16	3889302	0.078	10	Worcester Pearmain	0.141	0.019	15	NA	-0.068	NA	0.00031
Sor	SNP_FB_0337039	16	10594	chr16	1970558	Chr16	3891191	0.045	10	Worcester Pearmain	0.075	0.019	8	Jonathan	-0.046	0.154*	0.00010
Sor	SNP_FB_0337045	16	10597	chr16	1971603	Chr16	3892235	0.056	10	Worcester Pearmain	0.096	0.019	15	NA	-0.055	NA	0.00017

Supplementary Table S4. Significant loci detected by GWAS based on SNP genotypes (Supplementary Fig. S5).

Trait	Marker name	Chromosome	Order	GD Genome v1.0		GDDH13 Genome v1.1		Posterior prob. of QTL (γ)
				Chromosome	Position (bp)	Chromosome	Position (bp)	
PickDay	RosBREEDSNP_SNP_TC_27298641_Lg2_02271_MAF40_952256_exon1	2	1130	chr2	27298642	Chr02	26683848	0.667
PickDay	SNP_FB_0539966	3	1804	chr3	27701593	Chr03	26682124	0.265
PickDay	SNP_FB_0972401	3	1812	NA	NA	Chr03	26907044	0.561
PickDay	SNP_FB_0543925	3	1854	chr3	30726630	Chr03	25343333	0.620
PickDay	RosBREEDSNP_SNP_AG_31160644_Lg3_01779_MAF30_535055_exon1	3	1864	chr3	31160645	Chr03	30411691	0.982
PickDay	SNP_FB_0990635	5	2800	NA	NA	Chr05	42328332	0.320
PickDay	SNP_FB_0605906	5	2812	chr5	8171993	Chr05	41701481	0.368
PickDay	RosBREEDSNP_SNP_GT_3353551_Lg10_01947_MAF30_MDP0000950062_exon1	10	6030	chr10	3353551	Chr10	4150589	0.262
PickDay	RosBREEDSNP_SNP_GA_32372997_Lg10_RosCOS2362_MAF20_1643940_exon1	10	6583	chr10	32372998	Chr10	37809619	0.707
PickDay	SNP_FB_0063313	11	6842	chr11	7566331	NA	NA	0.268
PickDay	SNP_FB_0842249	11	6851	chr11	7692965	NA	NA	0.610
PickDay	SNP_FB_0151264	12	8052	chr12	33565531	Chr12	30067061	0.831
PickDay	SNP_FB_0285536	15	9782	NA	NA	Chr15	16834248	0.430
PickDay	RosBREEDSNP_SNP_GA_6845933_Lg16_02194_MAF50_390497_exon1	16	10771	chr16	6845933	Chr16	9128609	1.000
OvColor	RosBREEDSNP_SNP_TC_32540237_Lg9_01066_MAF50_787255_exon1	9	5873	chr9	32540239	Chr09	33587227	1.000
OvColor	SNP_FB_0239463	14	9079	chr14	20343500	Chr14	14952814	0.844
OvColor	SNP_FB_0286355	15	9812	chr15	16889886	Chr15	18257535	0.276
PerOC	SNP_FB_0811056	8	5251	chr9	27701751	Chr08	29610456	0.783
PerOC	RosBREEDSNP_SNP_GT_33056483_Lg9_01200_MAF50_MDP0000254312_exon19	9	5898	chr9	33056485	Chr09	34145319	0.964
PerOC	SNP_FB_0674147	14	9280	chr6	23188379	Chr14	25781799	0.327
RusTop	GDsnp01558	4	2510	chr4	21460139	Chr04	28516576	0.272
RusTop	GDsnp00619	4	2511	NA	NA	Chr04	28623344	0.774
RusBody	SNP_FB_0441434	1	457	chr1	30754868	Chr01	28687123	0.479
RusCal	RosBREEDSNP_SNP_CT_25680654_Lg2_01935_MAF40_403139_exon1	2	1099	chr2	25680655	Chr02	24760396	0.261
RusCal	SNP_FB_0660582	6	3664	chr6	11548046	Chr06	8149069	0.245
RusCal	SNP_FB_0014738	10	6077	chr10	10765679	Chr10	7837900	0.512
Scarf	SNP_FB_0764489	8	5209	chr8	32181256	Chr08	27662652	0.250
Scarf	SNP_FB_0764494	8	5212	chr8	32181761	Chr16	7105030	0.304
Scarf	SNP_FB_0067999	11	6929	chr11	11307225	Chr11	11172610	0.467
Scarf	SNP_FB_0867543	12	8073	chr12	34451508	Chr12	31051824	0.549
CraTop	SNP_FB_0336036	16	10581	NA	NA	Chr16	3479443	0.595
Drop	SNP_FB_0301373	15	9992	chr15	27881405	Chr15	26704476	0.338
Drop	RosBREEDSNP_SNP_AG_27882182_Lg15_00208_MAF30_MDP0000138089_exon7	15	9994	chr15	27882185	Chr15	26705256	0.311
Drop	SNP_FB_1069220	15	10019	chr14	29324904	Chr15	28941019	0.842
Drop	SNP_FB_0331634	15	10489	chr15	54145103	NA	NA	0.406
Juice	SNP_FB_0163853	13	8295	NA	NA	Chr13	4610915	0.170
Juice	RosBREEDSNP_SNP_CT_9996257_Lg16_00879_MAF50_1633349_exon1	16	11066	chr16	9996257	Chr16	22713840	0.412
Juice	SNP_FB_0366022	16	11090	chr16	21373337	NA	NA	0.159
WatCore	SNP_FB_0246451	14	9218	chr14	24795847	Chr14	24278265	0.328
WatCore	SNP_FB_0247146	14	9253	chr14	25515537	Chr14	24928023	0.322
Acidity	SNP_FB_0441434	1	457	chr1	30754868	Chr01	28687123	0.162
Acidity	RosBREEDSNP_SNP_AG_31626705_Lg1_00583_MAF40_MDP0000288458_exon4	1	470	chr1	31626705	Chr01	29526479	0.151
Acidity	GDsnp00639	8	4785	chr8	14519790	Chr08	7357008	0.266
Acidity	SNP_FB_0742398	8	4789	chr8	15005185	Chr08	7967180	0.467
Acidity	RosBREEDSNP_SNP_CT_17361806_Lg8_01370_MAF10_MDP0000220614_exon3	8	4874	chr8	17361807	Chr08	11027624	0.716
Acidity	SNP_FB_0050203	10	6630	chr10	36298025	Chr10	40085888	0.235
Acidity	SNP_FB_0918592	16	10571	NA	NA	Chr16	3186476	0.585
Acidity	RosBREEDSNP_SNP_AC_1452699_Lg16_MDP0000303483_MAF50_MDP0000303483_exon2	16	10573	chr16	1452699	Chr16	3304530	0.418
Weight	SNP_FB_0679078	5	3042	NA	NA	Chr05	28628478	0.252
Weight	SNP_FB_0825631	5	3367	NA	NA	Chr05	5626525	0.381
Weight	SNP_FB_0489512	11	7112	chr2	29535233	Chr11	27194200	0.386
Weight	SNP_FB_0160781	13	8240	chr13	2449242	Chr13	2826135	0.212
Weight	SNP_FB_0271888	15	9625	chr15	7328666	Chr15	10443896	0.367

Supplementary Table S4. (*Continued*)

Trait	Marker name	Chromosome	Order	GD Genome v1.0		GDDH13 Genome v1.1		Posterior prob. of QTL (γ)
				Chromosome	Position (bp)	Chromosome	Position (bp)	
Brix	SNP_FB_0953837	2	814	chr2	10699485	Chr02	9317521	0.111
Brix	GDsnp01223	2	815	chr2	10700457	Chr02	9318493	0.157
Brix	RosBREEDSNP_SNP_CT_29621172_Lg14_00171_MAF20_242588_exon1	14	9335	chr14	29621174	Chr14	28111084	0.193
Brix	RosBREEDSNP_SNP_GA_29623528_Lg14_00171_MAF20_521140_exon5	14	9336	chr14	29623530	Chr14	28113433	0.160
Brix	SNP_FB_0253401	14	9341	chr14	29659254	Chr14	28151776	0.111
Brix	SNP_FB_1069204	15	10018	chr14	29323079	Chr15	28942910	0.127
Brix	RosBREEDSNP_SNP_AG_44682057_Lg15_01805_MAF50_1633889_exon3	15	10341	chr15	44682060	Chr15	47685962	0.173
Brix	SNP_FB_0337042	16	10595	chr16	1970958	Chr16	3891590	0.129
Brix	SNP_FB_0337044	16	10596	chr16	1971223	Chr16	3891855	0.164
Brix	SNP_FB_0337051	16	10599	chr16	1973998	Chr16	3894629	0.111
Brix	SNP_FB_1075561	16	10756	NA	NA	Chr16	8848851	0.109
MalAcid	SNP_FB_0436932	1	396	chr1	26731420	Chr01	26510869	0.284
MalAcid	GDsnp01693	1	413	chr1	27104702	Chr01	26879708	0.179
MalAcid	GDsnp01822	3	1931	chr3	37542080	Chr03	35955492	0.242
MalAcid	SNP_FB_0748452	8	4886	chr8	17781132	Chr08	11610801	0.733
MalAcid	SNP_FB_1030996	8	4893	chr10	36809039	Chr08	11633374	0.190
MalAcid	SNP_FB_0073042	11	7004	chr11	14090592	Chr11	15434938	0.158
MalAcid	SNP_FB_0918592	16	10571	NA	NA	Chr16	3186476	0.510
MalAcid	SNP_FB_0918595	16	10572	NA	NA	Chr16	3186825	0.495
Firm	SNP_FB_0431170	1	322	chr1	21877737	Chr01	23578926	0.411
Firm	SNP_FB_0557658	3	1941	chr3	39872603	Chr03	36242799	0.510
Firm	SNP_FB_1046552	6	3751	NA	NA	Chr06	19407359	0.490
Firm	RosBREEDSNP_SNP_CT_16509379_Lg8_324685_MAF40_324685_exon1	8	4858	chr8	16509380	Chr15	6433343	0.302
Firm	RosBREEDSNP_SNP_TC_5075367_Lg11_RosCOS2981_MAF50_1677461_exon1	11	6778	chr11	5075367	Chr11	4282352	0.457
Firm	RosBREEDSNP_SNP_AC_5173582_Lg11_00418_MAF20_719249_exon1	11	6788	chr11	5173582	Chr11	4484758	0.450
DegMeal	RosBREEDSNP_SNP_CT_29946084_Lg3_01937_MAF50_1629511_exon2	3	1847	chr3	29946085	Chr03	29383600	0.090
DegMeal	SNP_FB_0543923	3	1853	chr3	30726426	Chr03	25343129	0.264
DegMeal	SNP_FB_0543932	3	1857	chr3	30728351	Chr03	25345071	0.135
DegMeal	SNP_FB_0545607	3	1865	chr3	31540046	Chr03	30823603	0.163
DegMeal	SNP_FB_0590716	4	2544	chr4	22741887	Chr04	29763289	0.220
DegMeal	SNP_FB_0986767	4	2551	chr4	22759964	Chr04	29768374	0.090
DegMeal	SNP_FB_0591255	4	2559	chr4	23444169	Chr04	30124476	0.239
DegMeal	SNP_FB_0631225	5	3132	chr5	25805458	Chr05	23163593	0.094
DegMeal	SNP_FB_0631226	5	3133	chr5	25805580	Chr05	23163471	0.338
DegMeal	SNP_FB_1063419	5	3137	NA	NA	NA	NA	0.290
DegMeal	RosBREEDSNP_SNP_CT_14298908_Lg8_RosCOS761_MAF40_MDP0000306801_exon2	8	4780	chr8	14298908	Chr08	7142498	0.123
DegMeal	SNP_FB_0027480	10	6380	chr10	19494741	Chr10	26267118	0.939
DegMeal	GDsnp01264	10	6456	NA	NA	Chr10	32165009	0.080
DegMeal	RosBREEDSNP_SNP_TG_3463798_Lg11_01167_MAF20_469664_exon1	11	6744	chr11	3463798	Chr11	2692887	0.087
DegMeal	SNP_FB_0270357	15	9610	chr15	6552025	Chr15	9626792	0.118
DegMeal	RosBREEDSNP_SNP_AC_6637447_Lg15_MDP0000156137_MAF20_MDP0000156137_exon1	15	9616	chr15	6637448	Chr15	9712213	0.395
DegMeal	RosBREEDSNP_SNP_CT_17586388_Lg15_RosCOS2945_MAF50_1674931_exon1	15	9793	chr15	17586390	Chr15	17544296	0.101
DegMeal	SNP_FB_0287539	15	9796	chr15	17551981	Chr15	17578711	0.093
MaxMeal	SNP_FB_0543923	3	1853	chr3	30726426	Chr03	25343129	0.205
MaxMeal	SNP_FB_0761428	8	5169	chr8	29887068	Chr08	26272598	0.250
MaxMeal	SNP_FB_0026018	10	6365	chr10	18871174	Chr10	25567242	0.207
Suc	SNP_FB_0455800	2	699	NA	NA	Chr02	5023450	0.120
Suc	SNP_FB_0493196	2	1180	chr2	32267290	Chr02	29933630	0.193
Suc	SNP_FB_0067998	11	6928	chr11	11306652	Chr11	11172037	0.217
Suc	SNP_FB_0068004	11	6930	chr11	11308610	Chr11	11173996	0.154
Suc	SNP_FB_0853797	11	7402	chr11	39347434	Chr11	42201133	0.177
Suc	SNP_FB_0528783	15	10219	chr3	15753588	Chr15	41722657	0.158
Suc	SNP_FB_0316403	15	10226	chr15	41911146	Chr15	42983163	0.131
Glu	SNP_FB_0553344	3	1913	chr3	37012969	Chr03	35354097	0.445
Glu	SNP_FB_0234418	6	3763	chr14	16825976	Chr06	24036606	0.193
Glu	SNP_FB_1096138	8	5129	chr5	32325259	Chr08	23727669	0.146
Glu	SNP_FB_1103580	8	5201	NA	NA	Chr08	27434639	0.141
Glu	SNP_FB_0853797	11	7402	chr11	39347434	Chr11	42201133	0.290
Glu	SNP_FB_0153836	12	8108	chr12	34959239	Chr12	31546712	0.669
Glu	RosBREEDSNP_SNP_GA_21188396_Lg17_00423_MAF50_MDP0000538106_exon1	17	11697	chr17	21188396	Chr17	28496419	0.742
Fru	SNP_FB_0761424	8	5168	chr8	29886791	Chr08	26272321	0.205
Fru	SNP_FB_0853797	11	7402	chr11	39347434	Chr11	42201133	0.245
Fru	RosBREEDSNP_SNP_GA_21188396_Lg17_00423_MAF50_MDP0000538106_exon1	17	11697	chr17	21188396	Chr17	28496419	0.208
Fru	SNP_FB_0406871	17	11759	chr17	25366876	Chr17	32632705	0.248
Sor	SNP_FB_1074691	16	10587	NA	NA	Chr16	3773426	0.196
Sor	RosBREEDSNP_SNP_TC_1972373_Lg16_00047_MAF40_480165_exon2	16	10598	chr16	1972373	Chr16	3893010	0.108
Sor	SNP_FB_0337051	16	10599	chr16	1973998	Chr16	3894629	0.218

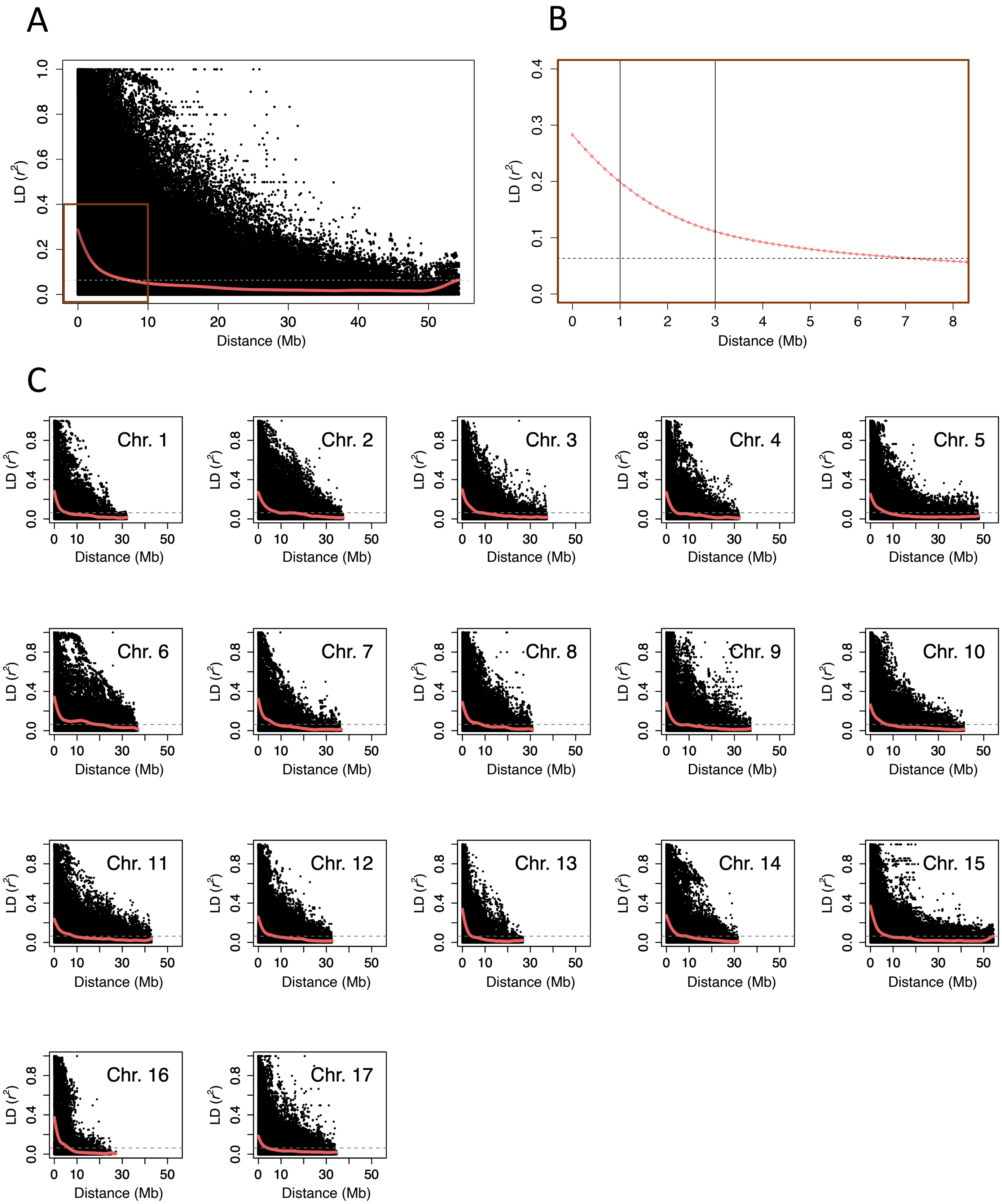

Supplementary Figure S1. LD values (r^2) between SNP pairs plotted against physical distances between the SNPs in the combined population.

(A) The entire genome. (B) The magnified image of the local area indicated by brown in (A) (r^2, 0–0.4; distance, 1–8 Mb). (C) Individual chromosomes.

Curves show local polynomial smoothed plots with kernel weight. Horizontal dashed lines correspond to the baseline r^2 values based on the 95-percentile of the distribution of r^2 values between pairs of unlinked markers.

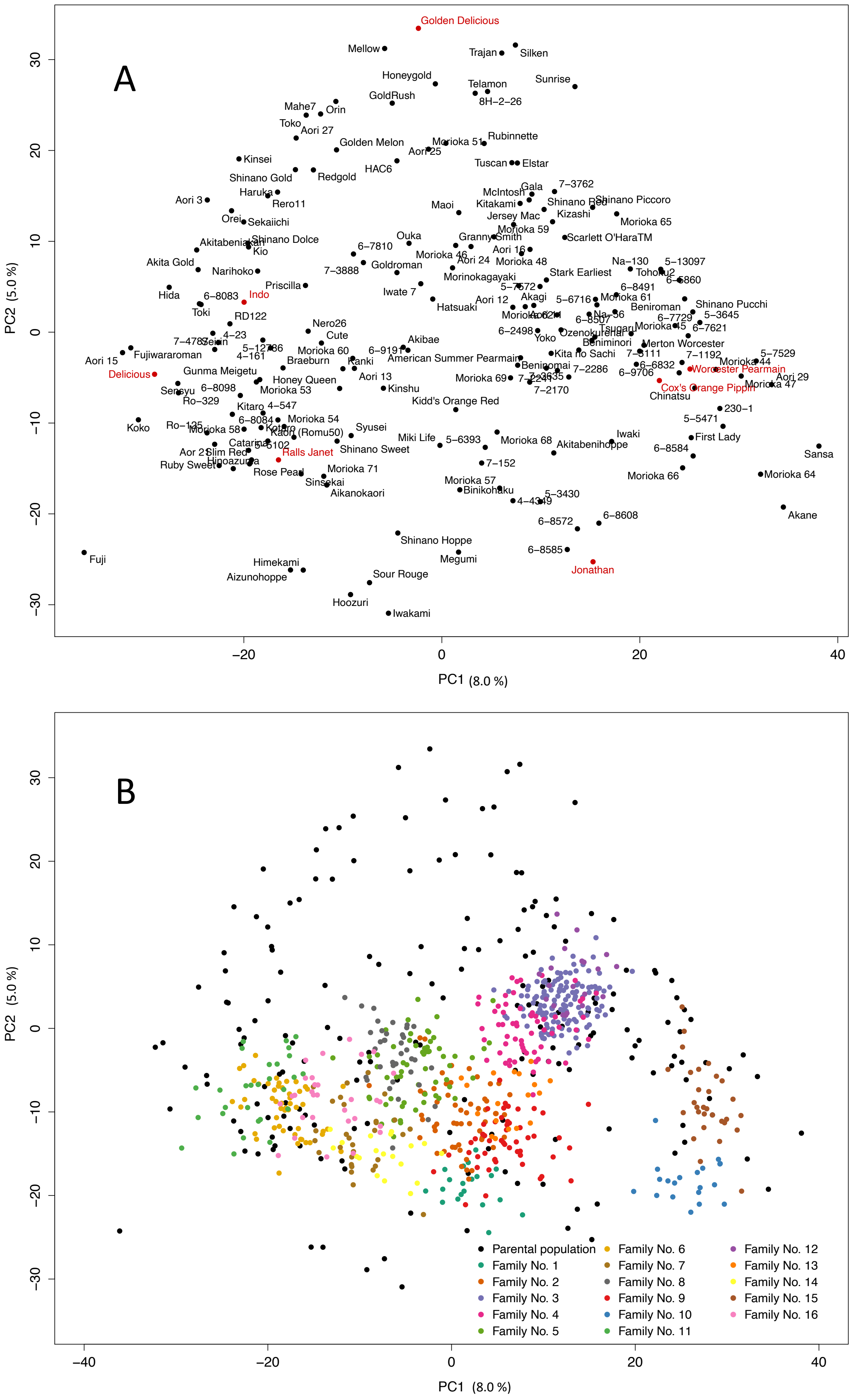

Supplementary Figure S2. Principal component analysis.

(A) Parental population; red indicates seven founder cultivars. (B) Combined population; black and colored circles indicate parental and breeding (Supplementary Table S2) populations, respectively.

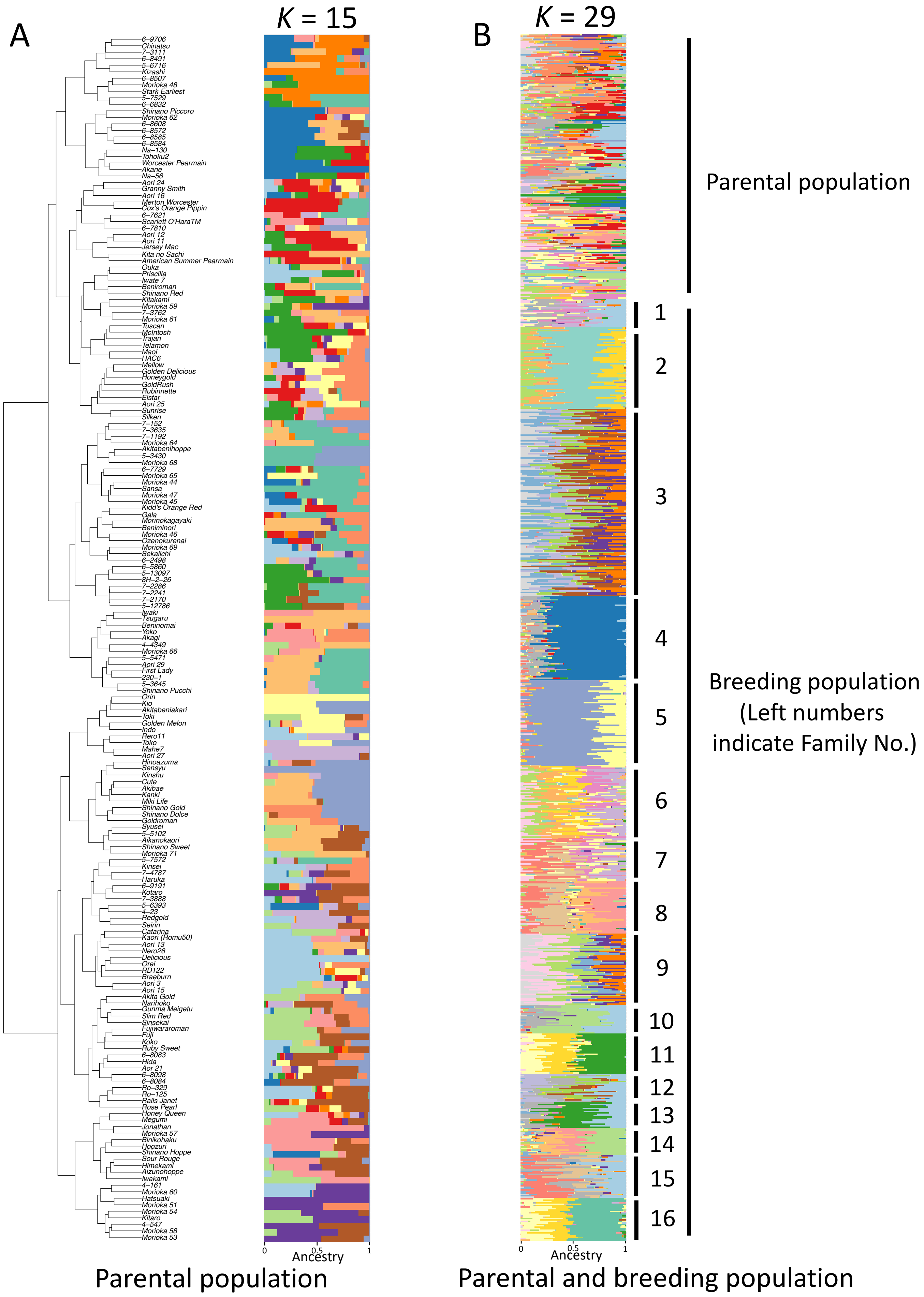

Supplementary Figure S3. Genetic population structure.

(A) Parental population; Ward's hierarchical clustering (left) and ADMIXTURE-based estimation of the admixture proportions of individuals (right). (B) Combined population; ADMIXTURE-based estimation.

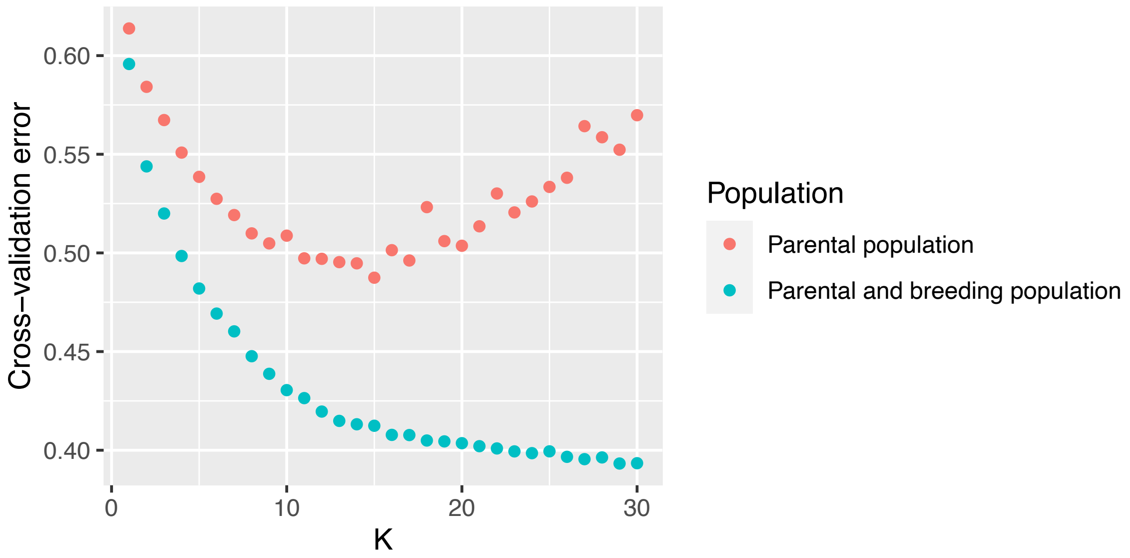

Supplementary Figure S4. Cross-validation errors returned by ADMIXTURE. K = 15 and K = 29 showed the lowest cross-validation errors in the parental population and the combined parental and breeding population, respectively.

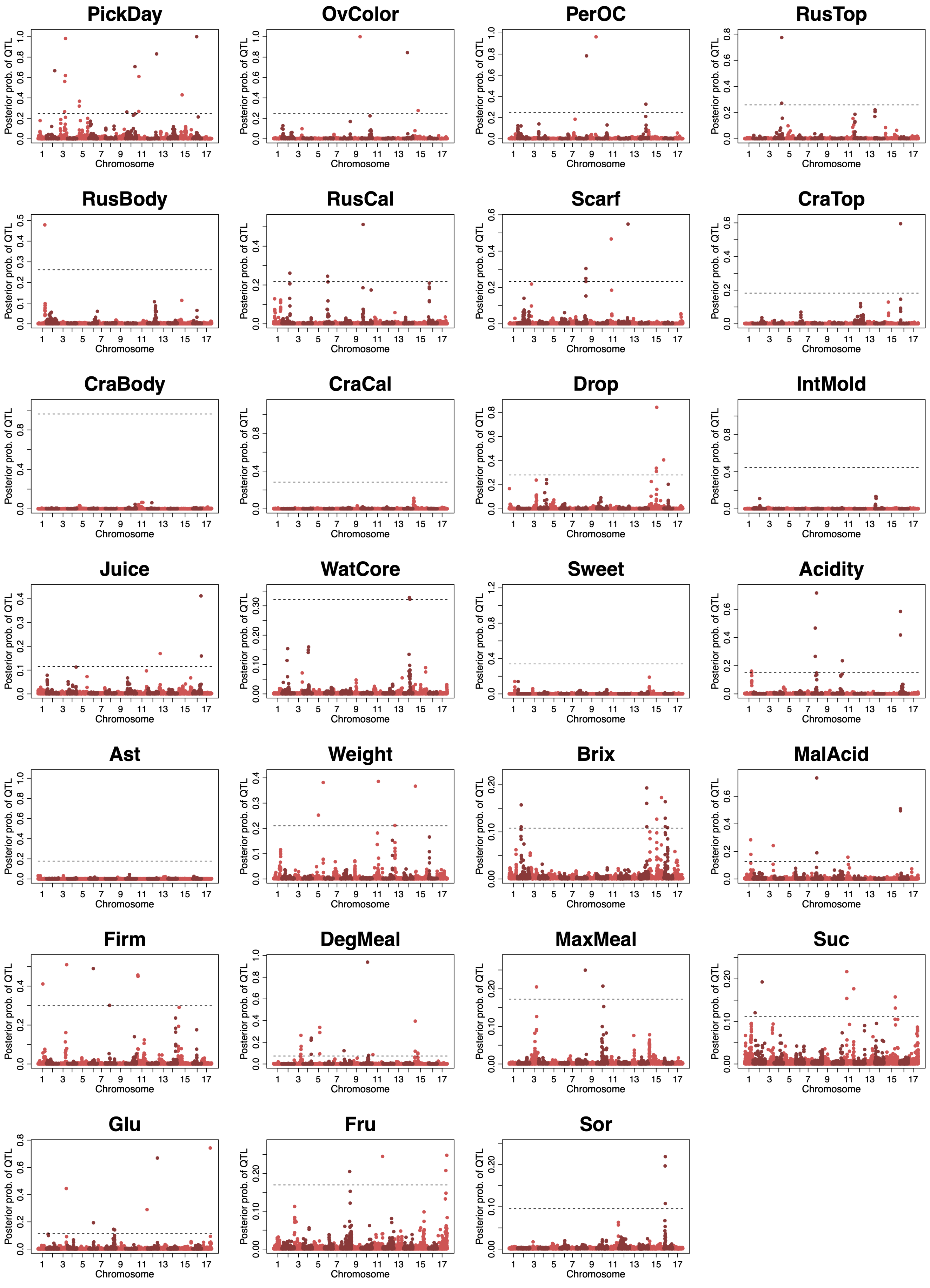

Supplementary Figure S5. GWAS based on SNPs in the combined population. Dashed lines indicate the significant threshold obtained from the random permutation analysis. Significant SNPs are listed in Supplementary Table S4.

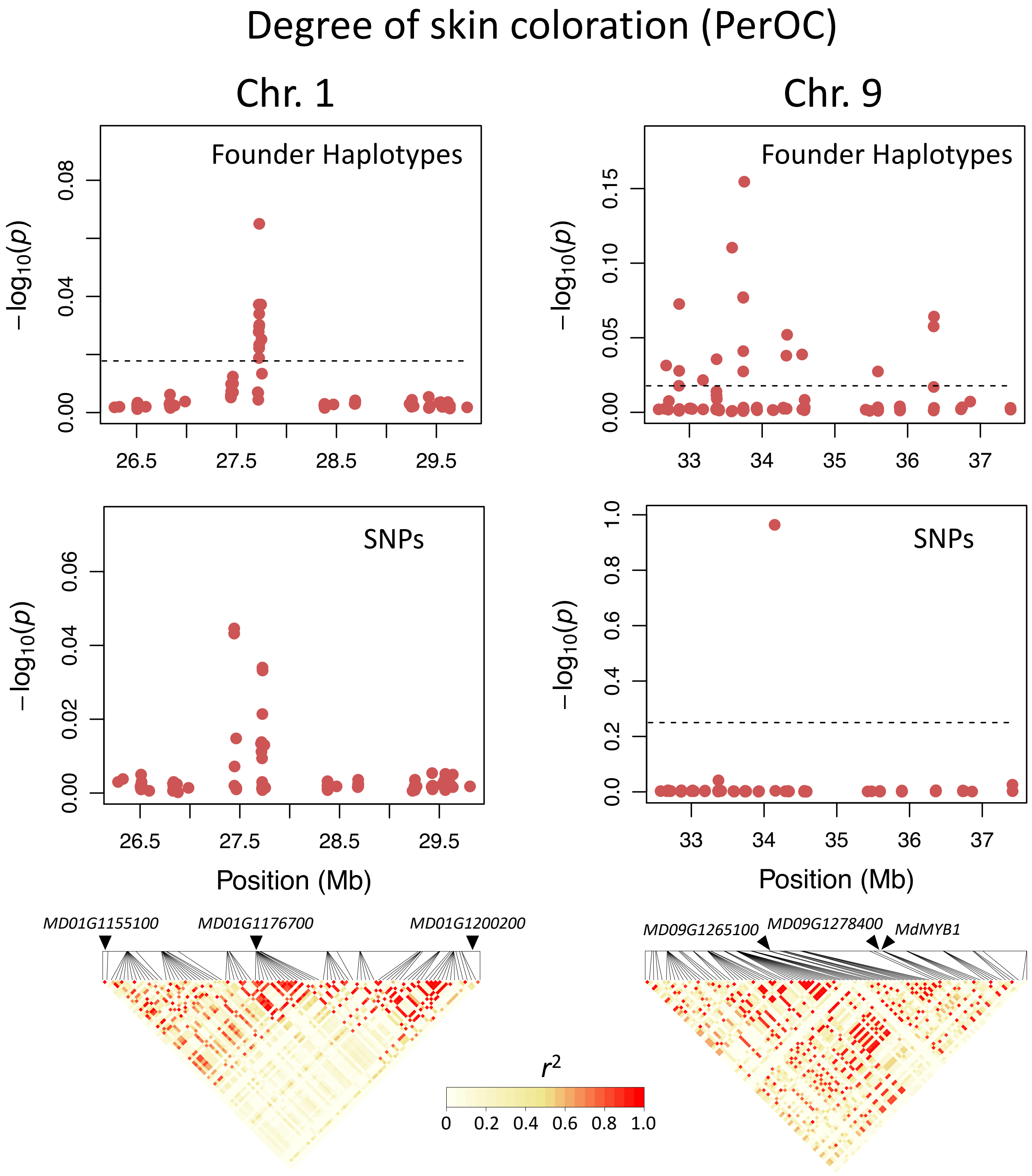

Supplementary Figure S6. Local Manhattan plots and LD heatmaps of the regions surrounding the peaks on chromosomes 1 and 9 for the degree of skin coloration (PerOC) in the combined population.

The dashed lines indicate the significance threshold obtained from the random permutation analysis. Significant SNPs are listed in Supplementary Tables S3 and S4. Arrowheads indicate the positions of the *MD01G1155100*, *MD01G1176700*, and *MD01G1200200* in Chr.1 and *MdMYB1*, *MD09G1265100*, and *MD09G1278400* in Chr. 9.

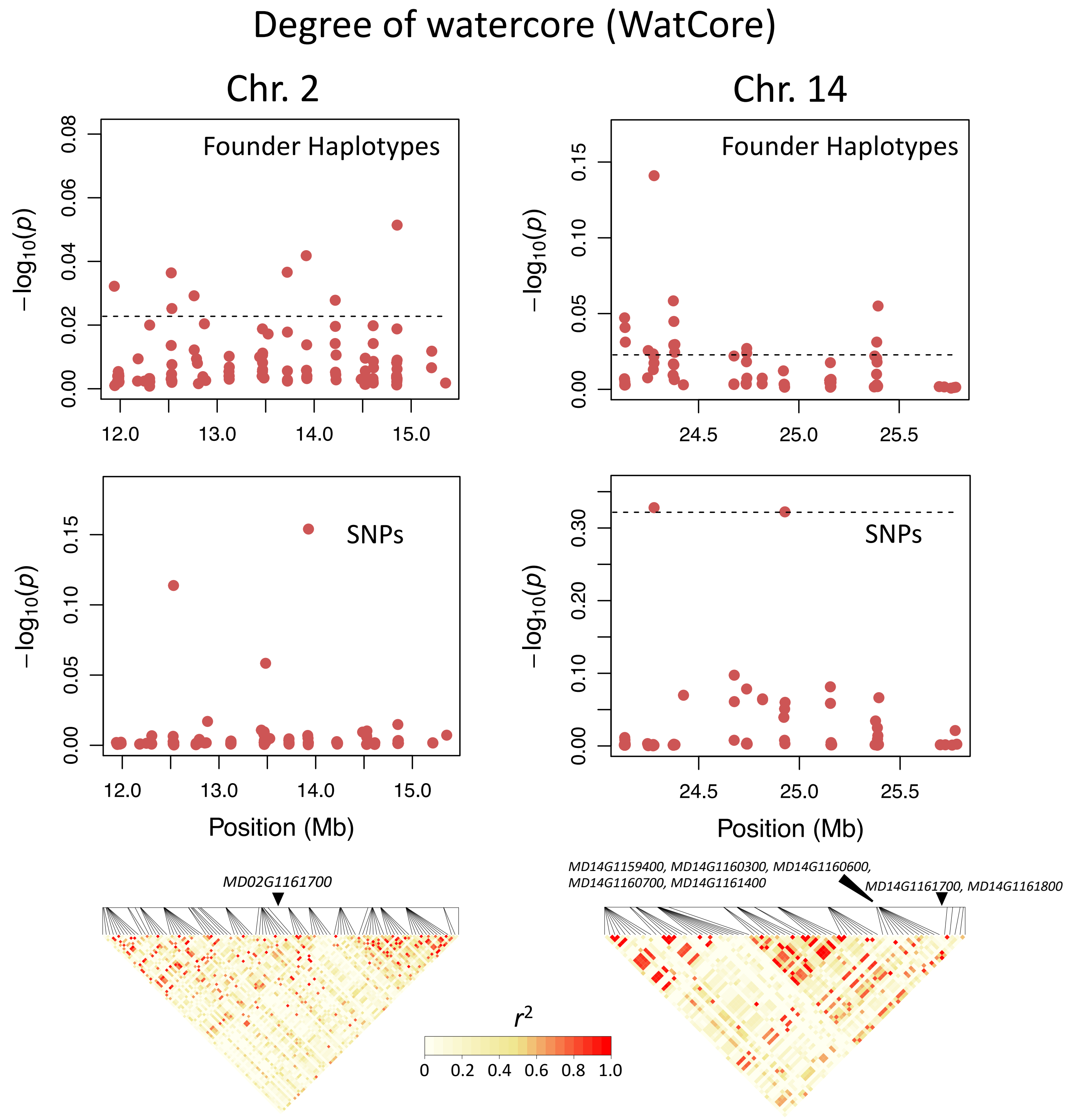

Supplementary Figure S7. Local Manhattan plots and LD heatmaps of the regions surrounding the peaks on chromosomes 2 and 14 for the degree of watercore (WatCore) in the combined population.
The dashed lines indicate the significance threshold obtained from the random permutation analysis. Significant SNPs are listed in Supplementary Tables S3 and S4. Arrowheads indicate the positions of *MD02G1161700* in Chr.2 and *MD14G1159400, MD14G1160300, MD14G1160600, MD14G1160700, MD14G1161400, MD14G1161700,* and *MD14G1161800* in Chr. 14.

Supplementary Methods

Contribution of the significant marker locus detected by GWAS with founder haplotypes

Based on Eq. 1 in Iwata *et al.* (2013), the contribution of the significant marker locus detected by GWAS with founder haplotypes on the phenotypic variance of the trait was calculated as follows:

$$a_{ij} = \sum_{l}^{L_j} \gamma_j \left(x_{ijl} + x'_{ijl}\right)\beta_{jl}$$

$$\mathrm{Var}[\boldsymbol{a_j}]/\mathrm{Var}[\boldsymbol{y}]$$

$\boldsymbol{a_j} = (a_{j1},\ a_{j2}, \dots, a_{jn})'$ and $\boldsymbol{y} = (y_1,\ y_2, \dots, y_n)'$ are the N-length vectors of the estimated effects of the significant marker j and the observed values, respectively, and where N is the number of individuals. L_j denotes the number of founder haplotypes in the significant marker j. $x_{ijl}(x'_{ijl})$ is an N × L design matrix, where L is the number of founder haplotypes in the significant marker j for variety i, and represents the composition of founder haplotypes in each individual. γ_j and β_{jl} are the posterior means of the probability of having a QTL and the genetic effect associated with the founder haplotype *l* for the significant marker *j*, respectively.

Reference

- Iwata, H. *et al.* Potential assessment of genome-wide association study and genomic selection in Japanese pear *Pyrus pyrifolia*. *Breed. Sci.* **63**, 125–140 (2013).
